# Phosphate Uptake and Allocation – A Closer Look at *Arabidopsis thaliana* L. and *Oryza sativa* L.

**DOI:** 10.3389/fpls.2016.01198

**Published:** 2016-08-15

**Authors:** Ewa Młodzińska, Magdalena Zboińska

**Affiliations:** Department of Plant Molecular Physiology, Institute of Experimental Biology, University of WrocławWrocław, Poland

**Keywords:** *Arabidopsis thaliana*, mycorrhiza, *Oryza sativa*, PHO1, phosphate translocators, phosphate transporters, phosphorus, transport

## Abstract

This year marks the 20th anniversary of the discovery and characterization of the two *Arabidopsis PHT1* genes encoding the phosphate transporter in *Arabidopsis thaliana*. So far, multiple inorganic phosphate (Pi) transporters have been described, and the molecular basis of Pi acquisition by plants has been well-characterized. These genes are involved in Pi acquisition, allocation, and/or signal transduction. This review summarizes how Pi is taken up by the roots and further distributed within two plants: *A. thaliana* and *Oryza sativa* L. by plasma membrane phosphate transporters PHT1 and PHO1 as well as by intracellular transporters: PHO1, PHT2, PHT3, PHT4, PHT5 (VPT1), SPX-MFS and phosphate translocators family. We also describe the role of the PHT1 transporters in mycorrhizal roots of rice as an adaptive strategy to cope with limited phosphate availability in soil.

## Introduction

Phosphorus is one of the macronutrients required by plants to grow and develop, but it is also one of the less accessible elements due to the very low solubility and poor mobility in soil solution as well as incorporation of phosphorus into organic compounds. It is estimated that about 20–80% of phosphorus in soils is present in organic matter mainly as phytic acid (Richardson, 1994; Schachtman et al., 1998). In addition, in acidic soils, phosphate forms insoluble precipitates with aluminum (Al) and iron (Fe), while in alkaline soils Pi is reactive with calcium (Ca) and magnesium (Mg; Raghothama, 1999; Hirsch et al., 2006). The primary source of phosphorus for plants is inorganic phosphate (Pi), and at neutral pH the predominant form H_2_PO_4_^-^ is transported into plant cells. Despite its quite low concentration in the soil solution (from 1 to 10 μM), the phosphate concentration in plant tissues is relatively high, about 5–20 mM (Raghothama, 1999; Hinsinger, 2001). This should not be surprising, because phosphorus is a fundamental element of essential biomolecules such as DNA, RNA, ATP, NADPH and membrane phospholipids. It also plays a crucial role for life-sustaining processes in plants including photosynthesis, respiration, and activation of proteins via phosphorylation (Poirier and Bucher, 2002). However, it was also reported that Pi concentration in the cytosol of *Arabidopsis thaliana* is only 60–80 μM (Pratt et al., 2009).

To cope with Pi limitations in the environment, plants have evolved a range of physiological and morphological responses, which may enhance Pi acquisition (through symbiotic strategies, root architectural changes, extrusion of organic acids and acid phosphatases by roots (reviewed by Amtmann et al., 2006; Péret et al., 2011; Zhang Z. et al., 2014; Scheible and Rojas-Triana, 2015) and optimize internal Pi utilization. During P limitation some membrane phospholipids are partially replaced by galactolipids and sulfolipids (Nussaume et al., 2010; Siebers et al., 2015) Furthermore, under Pi starvation stress the remobilization of phosphorus from older leaves to younger organs was observed (Smith et al., 2003).

A distinctive, visible symptom of Pi starvation is anthocyanin accumulation in many plants, which leads to purpling leaves, stems and even roots. It is known that anthocyanin production is induced by phosphorus and nitrogen deficiency, as well as by other environmental stresses (salinity, cold, high light intensity), but the regulation of anthocyanins under environmental stresses remains unclear. A recent report suggested that anthocyanins play an important role in photoprotection of light-harvesting proteins in photosystems (Henry et al., 2012). Ito et al. (2015) attributed purpling in *A. thaliana* to the strigolactone signaling pathway and proposed that strigolactones modulate anthocyanin accumulation under low Pi conditions.

Twenty years ago, discovery of two *Arabidopsis* Pi transporters was a milestone in the plant molecular biology of phosphate nutrition (Muchhal et al., 1996). Significant progress has been made in deciphering the mechanism underlying action of Pi transporter networks in specific tissues, cells and organelles in response to phosphorus deficiency (described by Rausch and Bucher, 2002; Péret et al., 2011, 2014; Baker et al., 2015). The molecular mechanism regulating the expression of genes encoding phosphate transporters and signaling pathways in wild and cultivated plants including *A. thaliana* and rice has been reviewed comprehensively in recent articles (Chiou and Lin, 2011). Previous studies have established the co-existence of high and low-affinity phosphate transport systems in the plants roots (Nussaume et al., 2010). The high affinity transporters are plasma membrane proteins that are responsible for Pi uptake from soil. These proteins are encoded by members of the *PHT1* (Phosphate Transporter) gene family and are proton-coupled H_2_PO_4_^-^ symporters. Movement of inorganic phosphate via PHT1 is driven by plasma membrane H^+^-ATPase (Ullrich-Eberius et al., 1981). From roots Pi is loaded into xylem via the PHO1 transporters, where Pi is allocated to the aerial parts of plants (Poirier and Bucher, 2002; López-Arredondo et al., 2014). Organic forms of Pi such as nucleotides (ATP) and hexose phosphates are transported in phloem sap (Rausch and Bucher, 2002). To maintain Pi homeostasis at the cellular level plants store Pi in the vacuole, and recently VPT1 (Vacuolar Phosphate Transporter 1) has been characterized as a tonoplast influx transporter (Liu et al., 2015). The importance of controlling Pi level also extends to other organelles, including plastids, mitochondria and Golgi. Phosphate transport across the plastid, mitochondrial and Golgi membranes is mediated by proteins of the PHT2/4 and, PHT3 and PHT4 families, respectively (Rausch and Bucher, 2002). All proteins involved in acquisition and translocation of Pi between cell compartments will be described in further detail below. Thus, the present work synthesizes a more complete knowledge about phosphate transporters with special attention to the role of these proteins in two species: the dicot model plant *A. thaliana* L. and the important crop, as well as, monocot model plant *Oryza sativa* L.

## Plasma Membrane Phosphate Transporters

**FIGURE 1:**
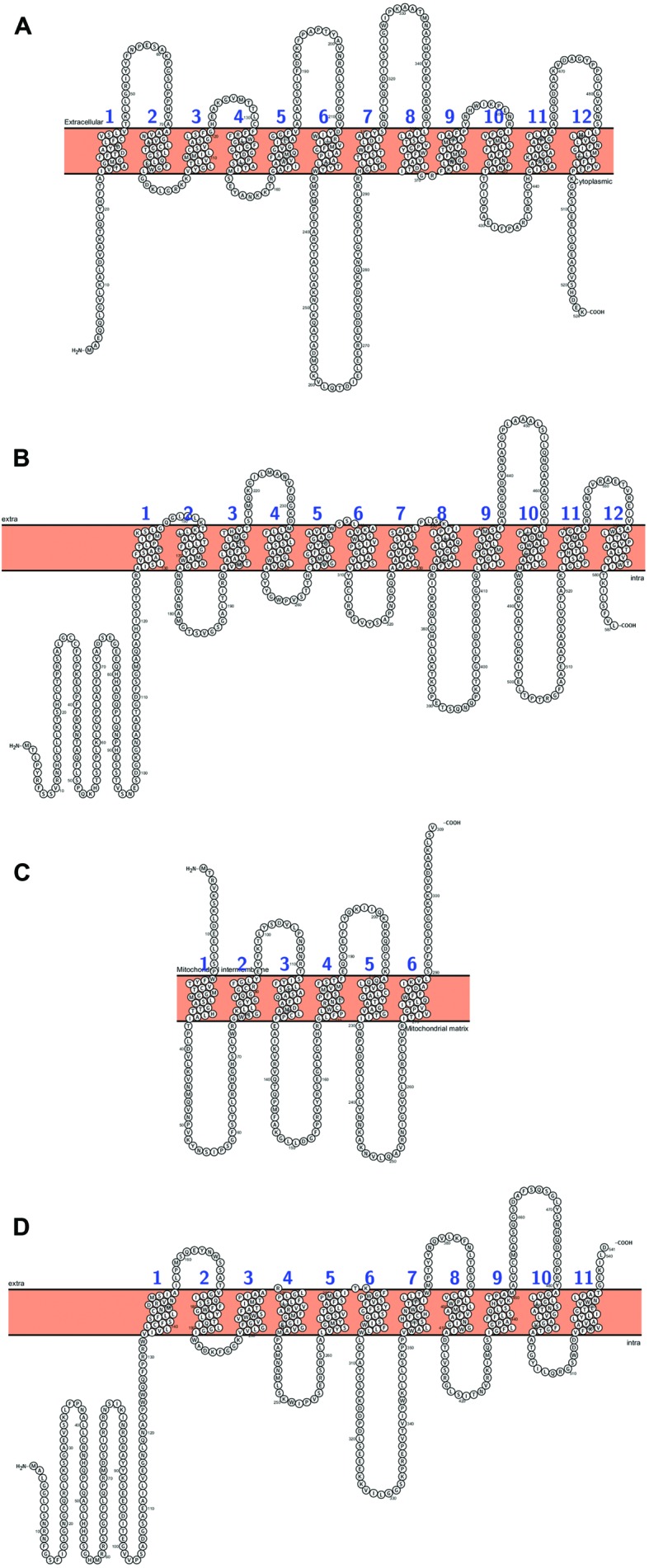
***Arabidopsis* PHT proteins predicted topology: (A) AtPHT1;1, (B) AtPHT2;1, (C) AtPHT3;3, (D) AtPHT4;4.** All figures were performed in Protter (Protter: interactive protein feature visualization and integration with experimental proteomic data. Omasits et al., 2014), protein sequences come from UniProt database.

As mentioned above, two types of transporters, PHT1 and PHO1, are responsible for phosphate uptake from soil and its further allocation to above-ground plant organs and between plant tissues. PHT1 proteins are the best described plant phosphate transporters. They belong to the family of phosphate: H^+^ symporters (PHS) within the major facilitator superfamily (MFS). The phosphate and proton transport stoichiometry is two to four H^+^ ions for each Pi. PHT1 transporters have been identified only in plant and fungal cells, and *in silico* analyses have established that their secondary structures share a common building plan, with 12 putative transmembrane domains (TM) separated into two groups by a large hydrophilic loop between TM6 and TM7. N- and C-termini of the proteins are oriented toward the cytoplasm (**Figure 1**; Raghothama, 1999; Poirier and Bucher, 2002; Rausch and Bucher, 2002; Liu et al., 2011; Nussaume et al., 2011). The crystal structure of PiPT, a fungal high-affinity phosphate transporter from *Piriformospora indica*, confirms the predicted Pht1 topology and demonstrates that the structure is conserved among plants and fungi (Pedersen et al., 2013). PHT1 transporters have been described in a wide range of plant species (list of plant PTH transporters and their main features – Supplementary Table S1), but they are best characterized for *A. thaliana* and *O. sativa*, whose genomes encode 9 and 13 PHT1s, respectively (Liu et al., 2011; Nussaume et al., 2011). *Pht1* expression is induced or strongly up-regulated during P-deprivation, but some *Pht1* genes are expressed regardless of the phosphate concentration in the environment (Mudge et al., 2002; Misson et al., 2004; Shin et al., 2004; Seo et al., 2008). It was also reported that PHT1 proteins can transport other solutes such as phosphite, arsenate, selenite, nitrate, sulfate, or chloride ions (Gu et al., 2016). Unfortunately, in many cases, functional analysis of particular PHT1 proteins is difficult because of their wide expression profile in most plant organs or complicated interpretation of results obtained from mutant analysis (discussed in more detail below).

**FIGURE 2:**
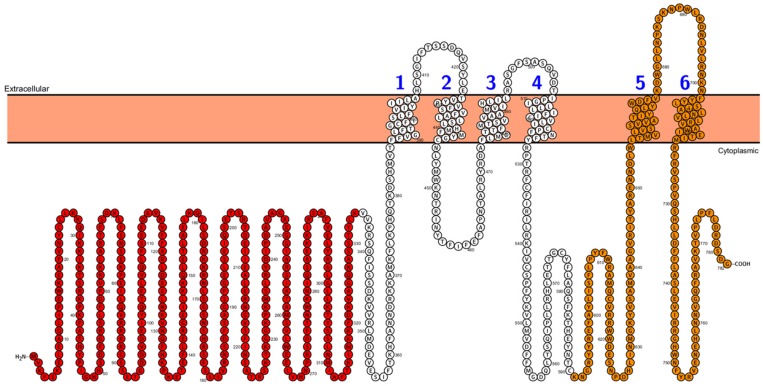
***Arabidopsis* PHO1 protein predicted topology.** Cytoplasmic N-terminus SPX domain is designed in red color, and the C-terminus with EXS domain is designed in orange color. Figure was performed in Protter (Protter: interactive protein feature visualization and integration with experimental proteomic data. Omasits et al., 2014), protein sequence comes from UniProt database.

Second type of transporters involved in phosphate distribution within the plant are PHO1 proteins. In contrast to PHT1, PHO1 belongs not to the MFS but to the SPX-EXS protein family (Secco et al., 2012). Beside transport properties PHO1 protein plays a role in signaling and participates in long distance (root-to-shoot) signal transduction cascade under Pi-deficiency. The topology of PHO1 from *A. thaliana* was recently analyzed – it consists of a long, cytoplasmic N-terminus harboring the tri-partite SPX domain, the cytoplasmic C-terminus with EXS domain and six membrane-spanning helices. The last two transmembrane helices are separated from the other four by a loop located in the cytoplasm and they are a part of the EXS domain (**Figure 2**). The EXS domain plays crucial role in PHO1 functioning – it is responsible for signaling capacity and Pi transporting activity of PHO1 (although it does not transport Pi by itself) as well as for proper protein localization in Golgi structures (Wege et al., 2016). PHO1 homologs are widely distributed in many organisms, including all land plants (bryophytes, lycophytes, gymnosperms, and angiosperms), fungi and animals (for example in *Drosophila*, *Caenorhabditis elegans* and mammals), but they have not been found in bacteria or in the unicellular green alga *Chlamydomonas reinhardtii* (Secco et al., 2012; Wege et al., 2016). Bioinformatics’ analysis revealed that the *Arabidopsis* genome has 10 *PHO1* homologs (*PHO1;H1–PHO;H10*), but to date the in Pi transport has been demonstrated only for PHO1 (Hamburger et al., 2002; Stefanovic et al., 2007, 2011; Arpat et al., 2012) and two PHO1 homologs, namely PHO1;H1 (Stefanovic et al., 2007) and PHO1;H3 (Khan et al., 2014). PHO1;H1 is able to compensate the loss of PHO1 function, whereas PHO1;H3 regulates PHO1 functioning under Zn-deficiency (Khan et al., 2014). The rice PHO1 family consist of three members: OsPHO1;1, OsPHO1;2 and OsPHO1;3, but only OsPHO1;2 seems to play a similar role to PHO1 from *A. thaliana* (Secco et al., 2010).

## Role of PHT1 in Direct Phosphate Uptake from Soil

Plants can take up phosphate directly from the soil or indirectly, form a mycorrhizal association, through the exchange of carbohydrates produced during photosynthesis for Pi released from fungus mycelium (Rausch and Bucher, 2002; Javot et al., 2007b). The direct strategy of phosphate uptake involves the PHT1 transporters present in the rhizodermis (especially in trichoblast cells) and, to a lesser extent, in the cortical cells. In *Arabidopsis*, eight of the nine *PHT1* are expressed in root tissues (Mudge et al., 2002; Shin et al., 2004; Lapis-Gaza et al., 2014), while in rice root the transcripts of all 13 *OsPHT1* were detected. Although, results obtained from different genetic experiments are sometimes discordant (Paszkowski et al., 2002; Seo et al., 2008; Yang et al., 2012), mutant analysis established that in phosphate uptake four transporters in *A. thaliana* (AtPHT1;1 – AtPHT1;4; Ayadi et al., 2015) and at least six PHT1 in *O. sativa* (OsPHT1;1/2/4/6/8/9/10; Seo et al., 2008; Ai et al., 2009; Jia et al., 2011; Wang X.F. et al., 2014; Ye et al., 2015) are principally involved.

Comprehensive analysis of *AtPHT1* genes expression in *Arabidopsis* roots under high Pi supply has shown the highest activity of *AtPHT1;1* promoter (Mudge et al., 2002). Nerveless, the transcripts of *AtPHT1;2*–*AtPHT1;4* were also detectable in the phosphate-fed roots. On the other hand, in Pi-deprived plants the *AtPHT1;1* mRNA level was only slightly enhanced (3- to 4-fold), while the expression of other genes from *AtPHT1* family increased much more distinctly (25- to 70-fold; Ayadi et al., 2015). Reporter genes (GUS or GFP) expression driven by the *AtPHT1;1* promoter revealed its localization in rhizodermis, especially in root hairs, but also in the lateral root caps, columella cells and, at relatively weak level, in cortical cells of the root hair zone (Mudge et al., 2002). Similarly, *AtPHT1;2* was expressed in trichoblast cells and in the cortex of older parts of root, the *AtPHT1;3* in trichoblasts of lateral roots and in epidermis near the root tip of the main root, as well as in pericycle cells of the main root (Mudge et al., 2002), while *AtPHT1;4* in the epidermis, cortex, stele, and root tips (Mudge et al., 2002; Misson et al., 2004). These results suggest that AtPHT1;1, AtPHT1;2, AtPHT1;3, and AtPHT1;4 may have similar, partially overlapping functions (Mudge et al., 2002), which was later confirmed experimentally (Misson et al., 2004; Shin et al., 2004; Ayadi et al., 2015). Namely, it was shown that under high Pi supply the phosphate uptake by the *atpht1;1* insertion mutant was reduced to around 60%, while in double mutant *atpht1;1Δ4Δ* to around 30% of the wild type rate (Shin et al., 2004). Such data suggests the main role of AtPHT1;1 in phosphate uptake by *Arabidopsis* roots at high Pi. Contrariwise, in P-starved plants, the predominant role in Pi acquisition plays AtPHT1;4, which is responsible for uptake of around 40–48% of Pi, while AtPHT1;1 contributes only in 15–20% of uptake and AtPHT1;2 and AtPHT1;3 together take up around 30% of Pi (Misson et al., 2004; Shin et al., 2004; Ayadi et al., 2015). Besides four main transporters (AtPHT1;2 – AtPHT1;4) the role of other AtPHT1 members in Pi acquisition cannot be ruled out. For example, the *atpht1;5* mutant exhibits moderate tolerance of arsenate (Pi structural analog, toxic for plants, which is transported via PHT), suggesting a possible role of AtPHT1;5 in Pi uptake (Nagarajan et al., 2011).

Reduced Pi uptake in knockout or knockdown (RNAi) lines and enhanced uptake in overexpressing lines has been demonstrated for rice OsPHT1 transporters (also named OsPT), including OsPHT1;1 (Sun et al., 2012), OsPHT1;2 (Liu et al., 2010), OsPHT1;4 (Ye et al., 2015; Zhang et al., 2015), OsPHT1;6 (Zhang F. et al., 2014), OsPHT1;8 (Jia et al., 2011), OsPHT1;9, and OsPHT1;10 (Wang X.F. et al., 2014). Among them only OsPHT1;1 is constitutively expressed, while the rest is remarkably upregulated under Pi deprivation (Seo et al., 2008; Wang X.F. et al., 2014). Only one protein from OsPHT1 family, the OsPHT1;2, is a low-affinity transporter (Ai et al., 2009). Interestingly, most of OsPHT1s do not exhibit the tissue-specific expression, but they are expressed ubiquitously in various root tissues and in some other plant organs. Therefore, it is reasonable to conclude that these transporters play probably a broad role in both, the phosphate acquisition and its translocation (Seo et al., 2008; Ai et al., 2009; Jia et al., 2011; Sun et al., 2012; Wang X.F. et al., 2014; Zhang F. et al., 2014; Ye et al., 2015; Zhang et al., 2015). Furthermore, tissues-specific expression of some *OsPHT1s* depends on developmental stage of plant. Good example is *OsPHT1;4* whose promoter activity was detected in all tissues of embryonic root (Zhang et al., 2015), while in mature plants growing under normal Pi it was found only in the exodermis – the outer layer of cortical cells which is involved in regulation of radial flow of water and ions in the root. During a long Pi-starvation, *OsPHT1;4* starts to be expressed in the cortical cells, which suggest its involvement in symplastic Pi transport under this stress condition (Ye et al., 2015). Taking into account all these data and the fact that overexpression or mutation of one of the *PHT1s* may alter the expression of the others (Jia et al., 2011; Lapis-Gaza et al., 2014; Ye et al., 2015), it is very difficult to ascertain which of these transporters are crucial for Pi uptake and distribution.

## Role of PHT1 in Phosphate Transport in Mycorrhizal Roots

It is estimated that 90% of terrestrial plant species can be colonized by mycorrhizal fungi (Smith and Smith, 2012) and more than 80% of vascular plants, including main crops, form arbuscular mycorrhizae (Karandashov and Bucher, 2005; Javot et al., 2007b). Whereas *A. thaliana* belongs to the minority of plant species that do not form mycorrhizal associations (Vance, 2008), *O. sativa* is an arbuscular mycorrhizal (AM) plant (Yang et al., 2012).

Arbuscular mycorrhizal fungi belong to the phylum *Glomeromycota*. Their hyphae penetrate the plant roots, enter cortical cells and inside each cell form hyphal coils or a heavily branched structure named an arbuscule (AM). Arbuscules or hyphal coils do not disrupt the integrity of the plant plasma membrane but through its invagination form intracellular spaces. Fungus infection alters the structure of cell plasma membrane allowing the growth of arbuscule/hyphal coils and creates a periarbuscular membrane (named the perihyphal membrane in the case of hyphal coil formation). The interface between this membrane and the hyphal plasma membrane is the place of nutrient exchange between symbiotic partners (Bucher, 2007; Javot et al., 2007b; Gu et al., 2011; Smith and Smith, 2012).

Beside other positive aspects of AM formation, such as protection against abiotic stresses and pathogens as well as improving of water acquisition from the soil, the best-known function of this type of mycorrhiza is the contribution of the fungus to plant P nutrition (Jeffries et al., 2003; Smith and Smith, 2012). On the other hand, Pi status of the plant is the main factor regulating the colonization process, delivery of sugars to hypha and symbiosis formation (Javot et al., 2007b; Gu et al., 2011). In plants inoculated with AM fungi two pathways of Pi uptake coexist. The first is the direct pathway from the soil through the membranes of root epidermis cells, involving the above-described PHT1 transporters. The second, indirect pathway via fungal mycelium engages plant PHT1 transporters located in the periarbuscular membrane at the arbuscule branch domain, which are specifically induced or upregulated by AM formation (Karandashov and Bucher, 2005; Kobea and Hata, 2010; Gu et al., 2011). Expression of these PHT1 transporters is closely correlated with the degree of root colonization and, at the single cell level, with the arbuscule formation and collapse (Paszkowski et al., 2002; Kobea and Hata, 2010). Physiological studies on several plant species (flax, tomato, rice, *Medicago truncatula*) have shown that under low Pi concentrations the mycorrhizal pathway is dominant (Yang et al., 2012). This is not surprising, because mycorrhizal formation is often accompanied with diminished expression of *PHT1* involved in the direct Pi uptake pathway (Javot et al., 2007b). Among rice *PHT1* genes, *OsPHT1;11* (Paszkowski et al., 2002; Kobea and Hata, 2010; Yang et al., 2012) and *OsPHT1;13* (Güimil et al., 2005; Yang et al., 2012) were proposed to be involved in the symbiotic Pi uptake route.

In fact, in most plant species forming mycorrhizal symbioses at least one mycorrhizal specific or upregulated *PHT1* gene has been discovered. What is more, phylogenetic analysis of PHT1 protein sequences showed that AM-associated PHT1s form their own lineage, which is evolutionarily distant from non-AM-associated PHT1s. Within this lineage several groups can be distinguished (Javot et al., 2007b; Yang et al., 2012). For example, OsPHT1;11 homologs are common among monocots and dicots and are evolutionarily older, being closely related to PHT1 transporters from the ancient plants *Physcomitrella patens* (bryophyte) and *Selaginella moellendorffii* (lycophyte), when OsPHT1;13 orthologs are conserved in monocotyledons, but not in dicotyledons (Yang et al., 2012).

*OsPHT1;11* and *OsPHT1;13* expression is undetectable in non-mycorrhizal roots but is induced after rice inoculation with *Glomus intraradices* or *Gigaspora rosea* (Yang et al., 2012). An increase of *OsPHT1;11* transcript level is significantly higher than *OsPHT1;13*, but the overall expression profiles of these two genes are similar. It was shown that promoters of both genes are specifically active in cortical cells containing arbuscules, although *OsPHT1;13*-*GUS* staining is weaker than *OsPHT1;11*-*GUS* staining. Mutation or downregulation of either *OsPHT1;11* or *OsPHT1;13* by RNAi causes strong reduction of fungal colonization and arbuscule development, but the phenotype of plants with altered expression of *OsPHT1;11* is definitely stronger. Interestingly, beside many similarities between these genes, their function in AM symbiosis establishment seems to be different. Mutations of both genes have no impact on Pi transport after mock inoculation, but uptake of ^33^Pi by *osph1;11* or *OsPHT1;11* RNAi lines inoculated with *G*. *intraradices* dramatically impaired relative to WT rice, whereas *osph1;13* and *OsPHT1;13* RNAi plants infected by AM fungus exhibit ^33^Pi acquisition similar to the uninfected plants. Moreover, expression of *OsPHT1;13* in yeast *pam2* mutant defected in Pi uptake did not complements yeast sensitivity to phosphate deprivation. These results strongly implicate that OsPHT1;13 operates as a mycorrhizal sensor rather than a transporter directly involved in Pi translocation through the periarbuscular membrane (Yang et al., 2012). Comparable sensor function was shown for AsPHT1;1 (AsPT1) from *Astragalus sinicus.* However, contrariwise to OsPHT1;13 this protein exhibits also transporting activity (Xie et al., 2013). Thus, it is highly feasible that both proteins (OsPHT1;13 and AsPHT1;1) can be classified as transceptors, the class of proteins combining receptor and transporter functions. As we know, during evolution some transceptor proteins may lose their transporting abilities (Popova et al., 2010; Gojon et al., 2011).

Although, some authors described *OsPHT1;11* and *OsPHT1;13* as genes specifically induced by mycorrhizal formation (Yang et al., 2012), other studies confirmed *OsPHT1;13* expression in the non-colonized roots subjected to low or high Pi supply (Glassop et al., 2007). Furthermore, comprehensive microarray analysis of *OsPHT1;13* expression in inoculated rice showed different levels of its transcript in vegetable and generative parts of in the rice plant at different lifecycle stages (Liu et al., 2011). The physiological functions of both proteins in the leaves and flowers of uninfected rice remain unclear, but this finding is not a rare occurrence. Maize *ZmPHT1;6*, *Brachypodium distachyon BdPHT1;7*, tomato *LePHT1;5* (Yang et al., 2012) and soybean *GmPHT1;7* (Inoue et al., 2014) genes specifically induced in roots by AM formation, are also expressed in above-ground plant organs as well as in asymbiotic roots.

## Role of PHT1 Transporters in Root Development

The impact of PHT1 on lateral root development has been proposed by several authors. Misson et al. (2004) observed strong *AtPHT1;4-GUS* activity in the central cylinder of the main root at the lateral root emerging point. At the beginning of the root branching, GUS staining was also strong in the central cylinder of lateral roots but later, during root emergence, it disappeared and, at the same time, evolved in the lateral root epidermis. Such expression profile was plausibly related to the role of AtPHT1;4 in Pi transport to the newly forming roots, and this process terminates when the young roots start to absorb phosphate from the soil (Misson et al., 2004). AtPHT1;4 seems, however, not to be crucial for the lateral root formation, as its mutation has no influence on this process. What is more, the local input of high Pi distinctly induced the lateral root elongation of the double *pht1;1Δ4Δ* mutant (Shin et al., 2004). The main function in development of lateral root in *O. sativa* has been proposed for *OSPHT1;2*, *OsPHT1;6* (Ai et al., 2009) and *OsPHT1;8* (Jia et al., 2011), according to their high promoter-GUS activities in the lateral root primordia.

In *A. thaliana*, overexpression of *AtPHT1;5* causes increased root hair density and length, and reduced primary root growth, irrespectively of the phosphate regimen (Nagarajan et al., 2011). Similarly, strong root hair proliferation under P-sufficient conditions occurred in *OsPHT1;1* overexpressing rice and in RNAi (*OsPHT1;1*-Ri) transgenic plants (Sun et al., 2012). These changes in root architecture were characteristic for P-starved plants and lead to the development of shallow but strongly branching root system with dense root hairs (Lambers et al., 2006). Above alterations in root architecture were perhaps not simply provoked by a low Pi concentration in roots but rather by the differential distribution of Pi between plant tissues/organs or by disturbances in the complex network of signaling pathways of P-deprivation, phytohormones, or other signaling molecules (Nagarajan et al., 2011; Sun et al., 2012). Similar, local modifications of lateral root formation could be promoted by patches rich in other nutrients, like nitrate and ammonium (Lima et al., 2010). Root system alterations in response to the local availability of mineral nitrogen forms were described for *atamt1;3* mutant with defected ammonium transporter (Lima et al., 2010) as well as for *atnrt1;1* mutant disrupted in nitrate transporter (Krouk et al., 2010). Experimental data indicated that in both cases the mutation affects the perception/signaling mechanism (Krouk et al., 2010; Lima et al., 2010). Furthermore, AtNRT1;1 participates directly in lateral root growth by regulation of auxin flow out of the primordium tip, which is dependent on the soil nitrate content (Krouk et al., 2010). Recent investigations have demonstrated the capability of the nitrate transporter (NRT) family members to transport other plant hormones (abscisic acid, gibberellin, jasmonoyl-isoleucine), so it seems to be a general characteristic of them (Chiba et al., 2015). As NRT, likewise PHT, belongs to the Major Facilitator Superfamily (Williams and Miller, 2001), it would be reasonable to investigate *pht1* mutants more precisely, in the context of root formation and phytohormones transport. Among PHT proteins, the role in Pi sensing and regulation of early lateral root branching was also proposed for two Pi transporters: MtPHT1;4 (MtPT4) and LjPHT1;4 (LjPT4). Expression of *MtPHT1;4* and *LjPHT1;4* genes occur in arbusculated cells in AM root. However, both transcripts were also found in the root tips of non-mycorrhizal roots. Expression impairment of all P-starvation marker genes in *mt/ljpht1;4* as well as *mt/ljtir1* mutants have might regulate the lateral root formation in similar manner as AtNRT1;1 transceptor (Volpe et al., 2016).

## Phosphate Allocation from Root to Shoot

**FIGURE 3:**
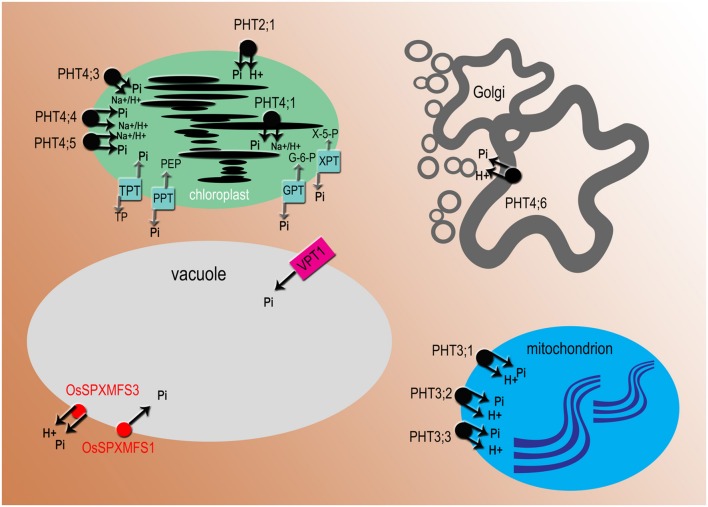
**Subcellular localization of phosphate transporters and translocators.** All names in black are for *Arabidopsis* proteins, in red for *Oryza sativa*. PHT2, PHT3, and OsSPX-MFS3 are proton-coupled Pi transporters, PHT4 transporters mediate H^+^ or Na^+^ dependent phosphate transport, VPT1 (PHT5;1) and OsSPX-MFS1 acts as Pi channel. PHT2, 3, 4 Phosphate Transporter 2, 3, 4; VPT1 (PHT5;1), Vacuolar Phosphate Transporter1 (Phosphate Transporter5;1); OsSPX-MFS, *O. sativa* (SPX)-Major Facility Superfamily. There are four types of Pi transloclators: TPT, triose-phosphate/phosphate translocator; PPT, phosphoenolpyruvate/phosphate translocator; GPT, glucose 6-phosphate/phosphate translocator; XPT, xylulose-5-phosphate translocator.

After uptake into the root cells Pi is subsequently used to synthesize P-containing compounds such as ATP or phospholipids, or can enter the vacuole, where it is stored (**Figure 3**). Nevertheless, the main fraction of phosphate is transferred toward the central cylinder, then released into xylem vessels and allocated to the stem, leaves, flowers, and seeds (Rausch and Bucher, 2002). At first, AtPHT1;9 and AtPHT1;8 were described as transporters involved in Pi uptake (Remy et al., 2012), but this hypothesis has been abandoned following results showing the role of both proteins in phosphate translocation from root to shoot, but not in phosphate acquisition directly from soil solution (Lapis-Gaza et al., 2014). It was also shown that these two proteins cooperate in phosphate translocation with PHO1 (Hamburger et al., 2002) and possibly with AtPHT1;3 and AtPHT1;4 (Mudge et al., 2002).

The *pho1* mutant of *A. thaliana* was first described in Poirier et al. (1991) as a mutant with disrupted Pi transfer into the xylem but normal phosphate uptake and its movement through the xylem system. Mutation of *PHO1* causes a strong P-starvation response with stunted growth and reduction of Pi content in shoots, to less than 5% compared to WT (Poirier et al., 1991). Later, the GUS method revealed that the *PHO1* promoter is active in the lower part of the hypocotyl, in the stele of roots (including xylem parenchymal cells and pericyclic) as well as in the endodermal cells adjacent to the protoxylem. Analogous to endodermis, pericyclic cells next to the protoxylem were stained more strongly than pericyclic cells farther away. No GUS staining was detected in the root tip or elongation zone (Hamburger et al., 2002).

*AtPHO1* expression in heterologous systems (yeast cells and *Xenopus* oocytes) have not confirmed the transporting activity of encoded protein (Hamburger et al., 2002). PHO1 transport activity was proved 9 years later, in *Arabidopsis PHO1* overexpressing lines, by Stefanovic et al. (2011). They showed that constitutive *PHO1* overexpression in leaves leads to more than 100-fold higher Pi concentration in guttation fluid (xylem sap), with a larger increase of Pi content (140-fold) in plants with stronger *PHO1* overexpression than in plants with weaker *PHO1* expression (100-fold increase; Stefanovic et al., 2011). A similar result, indicating the transporting role of PHO1, was obtained using *Arabidopsis* transgenic plants with *PHO1* under the control of an estradiol-inducible promoter (Arpat et al., 2012). All considered, the PHO1 subcellular localization results were rather surprising: PHO-GFP predominantly co-localized with the Golgi/*trans*-Golgi (Arpat et al., 2012), endoplasmic reticulum, and occasionally with endosomal markers (Liu et al., 2012). Moreover, this localization was associated with Pi export activity of PHO1. Nevertheless, in some circumstances, including a high Pi concentration in the cytoplasm, PHO1 could be partially redistributed to the plasma membrane, which leads to the conclusion that PHO1 relocalization between cell membranes is an element of the phosphate homeostasis sustaining system (Arpat et al., 2012). Likewise, the dual localization profile, in the plasma membrane and ER, exhibits for example the K^+^ channel TPK4 (Sharma et al., 2013). Other possibility is PHO1 engagement in Pi loading into endosomal vesicles and Pi export via exocytosis (Arpat et al., 2012).

The second PHO1 protein involved in Pi loading into the xylem is PHO1;H1. *PHO1;H1* is expressed in the vascular system of root and shoot, and is able to compensate the loss of *PHO1*. Although *pho1;h1* does not express any P-starvation hallmarks, the double mutant *pho1/pho1;h1* shows stronger phenotypic features than *pho1*, indicating that both proteins are involved in Pi influx into the xylem. Interestingly, *PHO1* and *PHO1;H1* seem to be regulated in various ways what may be connected with *Arabidopsis* flexibility during P-deficient stress (Stefanovic et al., 2007). Other *Arabidopsis PHO1* genes whose transcripts are localized in the root tissues were *PHO;H4* and *H10* in epidermal and cortical cells, *H5* and *H7* in the root tip, and *H3*, *H3*, *H5*, *H7*, and *H8* in the vascular cylinder (Wang et al., 2004). Among three rice PHO1 proteins only OsPHO1;2 plays a role similar to PHO1, and *ospho1;2* mutation leads to the reduction of phosphate content in shoot, phosphate over-accumulation in root tissues, and altered growth of plants (Secco et al., 2010).

*AtPHT1;8* and *AtPHT1;9* are both expressed in roots, more strongly during P-deficient than in P-replete conditions (Misson et al., 2004; Shin et al., 2004; Remy et al., 2012; Lapis-Gaza et al., 2014). *Atpht1;8* and *atpht1;9* mutant lines are characterized by the same uptake rate as WT plants and much slower short-term Pi accumulation in the shoot than *atpht1;1* plants. Moreover, they are expressed mainly in the endodermis and xylem of the meristematic zone and throughout the root in xylem pole pericycle cells. These observations suggest that AtPht1;8 and AtPHT1;9 might regulate Pi translocation into the xylem. It is also proposed that in the meristematic region AtPHT1;9 works in the root layer closer toward the epidermis, probably in the endodermis and/or pericycle, whereas AtPHT1;8 acts deeper, in pericycle or xylem cells (Lapis-Gaza et al., 2014). Taking into account that *AtPHT1;3* shows an expression pattern partially similar to *AtPHT1;8* and *AtPHT1;9* in pericycle cells along the root (Mudge et al., 2002), and that *AtPHT1;4* can be expressed in the stele of the main root at the secondary root branching points (Misson et al., 2004), probably in the cells interior to the pericycle strand (Mudge et al., 2002), it can be suggested that AtPHT1;3 and AtPHT1;4 may help AtPHT1;8 and AtPHT1;9 to play their roles. On the other hand, these transporters can potentially have a scavenging role and reabsorb Pi leaking from xylem vessels (Mudge et al., 2002). Additionally, as has been suggested for AtPHT1;4, they may participate in the lateral root emergence (Misson et al., 2004).

Among rice PHT1 members, OsPHT1;2 was chiefly proposed to mediate Pi transfer into the root vasculature, based on its low affinity to phosphate and spatial localization in the central cylinder of the lateral and main roots, but not in the endodermis, cortex, or epidermis. Reduced phosphate content in the shoot of *OsPHT1;2*-RNAi transgenic plants (Ai et al., 2009), and definitely higher Pi concentration in the shoots than in the roots of *OsPHT1;2* overexpressing line under P-sufficient conditions (Liu et al., 2010), compared to wild type, support this conclusion. Later research focused on the function of OsPHT1;2 in selenite uptake, providing evidence that *OsPHT1;2* mRNA is present in the rhizodermis of primary roots under P-deprived conditions. This discovery could be explained by developmental stage-depending expression of this gene, or by differences between rice cultivars used for in the study, but may also underline the additional role of OsPHT1;2 in Pi uptake (Zhang L. et al., 2014). *OsPHT1;2* is probably supported by other *OsPHT1* members, expression of which was detected in vascular tissue in the root: *OsPHT1;1* (Sun et al., 2012), *OsPHT1;4* (Zhang et al., 2015), *OsPHT1;6* (Ai et al., 2009; Zhang F. et al., 2014) and *OsPHT1;8* (Jia et al., 2011). Functional analysis of rice overexpressing *OsPHT1;1* showed no significant differences in Pi translocation to the shoot when rice was growing at a low Pi concentration, but under high Pi supply plants accumulated nearly two times more Pi in the xylem sap of stems (Sun et al., 2012). Respectively, higher or lower ^33^Pi concentration in xylem sap, under both P-deficient and P-sufficient conditions, was also observed in *OsPHT1;4* overexpressing, *ospht1;4* and *OsPHT1;4* RNAi rice. These plants were also characterized by altered shoot/root content of ^33^Pi (Zhang et al., 2015). Similarly, monitoring of ^33^P-labeled Pi distribution demonstrated a higher shoot to root ratio of ^33^P in the plants overexpressing *OsPHT1;6* (Zhang F. et al., 2014) or *OsPHT1;8* (Jia et al., 2011), indicating their role in phosphate allocation to the shoot. Nevertheless, we have to take into account that the observed changes in Pi distribution between root and shoot in transgenic plants may be caused by enhanced or defective uptake of Pi from nutrient solution.

## Phosphate Distribution Within Vegetative Organs

Co-ordinated phosphate distribution between plant organs and tissues is crucial to maintain Pi-homeostasis, indispensable for continuous growth and development. Pi is allocated from the root to the shoot via xylem, but during leaf senescence or nutritional phosphorus deprivation it is mobilized from old leaves and transported via phloem to the sink organs (young leaves, growing roots, flowers, or seeds). After xylem or phloem unloading, Pi is subsequently transferred to the surrounding cells (Poirier and Bucher, 2002; Rausch and Bucher, 2002). Pi remobilization is a highly efficient process: in *Arabidopsis* up to 78% of phosphorus from senescing leaves is remobilized in this way (Nagarajan et al., 2011), and in Australian *Hakea prostrata* a minimum of 85% of Pi (Shane et al., 2014). In Pi release from organic compounds in senescing organs participate primary purple acid phosphatases (PAPs) and RNases, whose activity inside the cells and in the cell walls increases dramatically in these conditions. RNA may contain up to 60% of organic phosphorus in mature leaves, and its disruption by RNase causes the release of free nucleotides, which in consequence may become PAP substrates (Shane et al., 2014). Thus, although phosphorus may also be transported in the phloem sap in organic form (as hexose-phosphate or nucleotides), Pi constitutes the main form of P in the phloem (Rausch and Bucher, 2002). In Pi distribution and recycling several rice and *Arabidopsis* PHT transporters are engaged.

Based on the expression pattern and mutants characteristics, it is presumed that *A. thaliana* phosphate transporter AtPHT1;5 plays a broad role in Pi redistribution between vegetative organs (Mudge et al., 2002; Nagarajan et al., 2011; Smith et al., 2011). Strong *AtPHT1;5* promoter activity was detected in the cotyledons and hypocotyl of young *Arabidopsis* seedlings, suggesting a role of AtPHT1;5 in remobilization of Pi released from phytate (see next section) to developing organs (Mudge et al., 2002). In this process *AtPHT1;5* may be supported by *AtPHT1;9* expressed in the seedlings (Remy et al., 2012) and *AtPHT1;1*, whose promoter activity was detected in the peripheral layer of the endosperm (Mudge et al., 2002).

Mudge et al. (2002) found that after a few days following germination, *AtPHT1;5* expression in the seedling drops, except cotyledons. Furthermore, in older plants *AtPHT1;5-GUS* labeling is restricted to the phloem cells of senescing leaves and flowers sepals. These findings gave credence to the notion that AtPHT1;5 is involved in the Pi redistribution process (Mudge et al., 2002). This conclusion is in agreement with later studies of *AtPHT1;5* overexpressing *Arabidopsis*. These plants displayed premature senescence, reduced ^33^Pi accumulation in older leaves and increased ^33^Pi content in siliques as well as increased transcript levels of RNase and phosphatase in rosette leaves (older leaves). Moreover, mutation or overexpression of *AtPHT1;5* led to altered Pi distribution between root and shoot; thereby under high Pi supply the mutant lines accumulate less P in roots, but more in shoots, when *AtPHT1;5* overexpressors have higher root/shoot total P content in relation to the WT. Because *AtPHT1;5* expression in shoot tissues is more intense when plants grow in high Pi supply, it is plausible that *AtPHT1;5* function in these conditions involves re-translocation of phosphate from the shoot back to the root. However, under P-deficient stress the *AtPHT1;5* promoter is active in the stele of the root, and in *atpht1;5* plants ^33^Pi allocation to the shoot is up to 40% lower, indicating the role of AtPHT1;5 in xylem loading (Nagarajan et al., 2011). Generally, to sum up all the findings described above, we can conclude that, depending on Pi concentration in the environment and developmental cues, AtPHT1;5 may play a role in: Pi loading into the root xylem, Pi mobilization from senescing leaves and sepals, Pi re-translocation to the root, and Pi transport to the seedling and young growing organs (Mudge et al., 2002; Nagarajan et al., 2011; Smith et al., 2011). What is important, changes in *AtPHT1;5* expression corresponded to differences in mRNA levels of *PHO1* as well as to the P-starvation response modulators (*miR399d* and *At*) which highlights the significance of this transporter for Pi homeostasis (Smith et al., 2011).

Other *Arabidopsis* transporters which may participate in P translocation in the shoot are AtPHT1;4 and AtPHT1;9. Apart from roots, among vegetative organs, the *AtPHT1;9* transcript was found in the seedlings and senescing leaves, but not in the stem or young, adult or cauline leaves, so it may indicate that, similar to AtPHT1;5, AtPHT1;9 is involved in the Pi mobilization (Remy et al., 2012). *AtPHT1;4* expression was detected in cotyledons, axillary buds, leaves, apical meristem and trichomes (Mudge et al., 2002; Misson et al., 2004). Furthermore, *AtPHT1;4* mRNA level in the leaves of plants growing in Pi-depleted and Pi-replete medium is similar to or even higher than the level of *AtPHT1;5* transcript, but no specific role of AtPHT1;4 in leaves was determined (Nagarajan et al., 2011).

Promoter activity of four *Arabidopsis PHT* genes (*AtPHT1;1*, *AtPHT1;3*, *AtPHT1;4*, and *AtPHT1;6*) has been detected in the hydathodes of leaves and cotyledons (Mudge et al., 2002). It was described that guttation droplet composition of both organic (Pilot et al., 2004) and inorganic (Nagai et al., 2013) solutes is different from that of xylem sap, which implicates secretion or retrieval of nutrients from hydathodes. Thus, as was pointed out by Mudge et al. (2002), we may presume that expression of phosphate transporters in hydathodes is due to their contribution in Pi absorption from guttation fluid, which prevents Pi loss (Mudge et al., 2002). Based on expression in hydathodes, similar function might be attributed to other *A. thaliana* transporters, such as the K^+^ channel AKT1 (Lagarde et al., 1996), sulfate transporters Sultr1;1 (Takahashi et al., 2000) and Sultr1;2 (Shibagaki et al., 2002), the nitrate transporter AtNRT2;1 (Nazoa et al., 2003) and the purine transporter AtPUP1 (Bürkle et al., 2003), while the GDU1 transporter is involved in glutamine excretion by hydathodes (Pilot et al., 2004). In fact, investigations of Nagai et al. (2013) clearly showed that potassium, nitrate and phosphate content in guttation fluid is lower, while chloride is higher, than concentrations of these ions in the xylem vessels close to hydathodes. Furthermore, Pi concentration in guttation droplets is almost zero, and an elegant experiment with tracing of ^32^P by autoradiography demonstrated conclusively that ^32^Pi can be taken up by hydathodes and redistributed to the whole plant within 1 day (Nagai et al., 2013).

It should also be noted that several *AtPHO1* homologs are expressed in *Arabidopsis* leaves and stem: *H10* across leaves, *H1* and *H3* in leaf vascular tissues, *H4*, *H7*, *H8* and *H10* in hydathodes, *H8* in trichomes, *H5*, *H7*, and *H10* across the stem, and *H1* and *H8* in the vascular cylinder of the stem (Wang et al., 2004). Nevertheless, these expression results are not necessarily related to *AtPHO1* involvement in Pi transport. The functions of major AtPHO1 proteins are unknown, and some of them play roles other than Pi translocation. For example, AtPHO1;H4 is localized in the nucleus and controls hypocotyl elongation in response to blue light, whereas AtPHO1;H10 is involved in stress responses (cold, salt, pathogens, etc.; Baker et al., 2015).

It was proposed that in the root Pi redistribution may be accomplished by AtPHT1;8 and AtPHT1;9, despite their functioning in xylem loading. *AtPht1;8* and *AtPht1;9* transcripts are detected in metaphloem, protophloem and phloem companion cells but only in the meristematic region. AtPht1;8 and AtPht1;9 presence in this root zone is presumably correlated with controlling Pi redistribution between shoot and root by phloem sap, and thus probably the phloem unloading process (Lapis-Gaza et al., 2014). Alternatively, if AtPht1;8 and AtPht1;9 activity will be detected in the upper part of the root phloem, we will be able to assign them a function similar to AtNRT1;9 nitrate transporter. This protein expressed in root companion cells is responsible for phloem loading and therefore the downward transport of nitrate in roots, to regulate NO_3_^-^ distribution in response to environmental changes (Wang and Tsay, 2011).

In rice leaves expression of *OsPHT1;1* (Seo et al., 2008; Sun et al., 2012), *OsPHT1;2* (Ai et al., 2009), *OsPHT1;4* (Ye et al., 2015), *OsPHT1;6* (Ai et al., 2009), *OsPHT1;8* (Jia et al., 2011; Li et al., 2015), *OsPHT1;9*, *OsPHT1;10* (Wang X.F. et al., 2014) and *OsPHT1;13* (Liu et al., 2011) was detected. The cross-section of the leaf blades of transgenic plants expressing the *GUS* gene under the control of *OsPHT1* promoters, indicating that promoters of almost all genes (except *OsPHT1;4* and *OsPHT1;13*, for which data about tissue-specific expression in the leaves are not available) are preferentially active in mesophyll, phloem and xylem, whereas their activity in epidermal cells is low (Ai et al., 2009; Jia et al., 2011; Sun et al., 2012; Wang X.F. et al., 2014). This expression pattern may be related to Pi mobility. It was shown that when other ions such as Na^+^, K^+^, Ca^2+^, NO_3_^-^, and Cl^-^ accumulate to the greatest extent in epidermal tissue, Pi concentrates in mesophyll cells, which probably favors quick Pi transfer to the phloem system during phosphate deprivation or leaf senescence (Nagai et al., 2013).

It was suggested that in Pi translocation from older leaves to sink organs OsPHT1;4 (Ye et al., 2015), OsPHT1;6 (Zhang F. et al., 2014) and OsPHT1;8 (Li et al., 2015) participate, but we cannot exclude the direct involvement of other rice Pi transporters, because of limited information. *OsPHTT1;4* is intensively expressed in the flag leaf but also in roots, culm, ligule, and generative organs. Its expression increases under limiting Pi conditions and changes during rice development, reaching a peak in flag leaves after pollination. Flag leaves are the key source of Pi for developing seeds, and *OsPHT1;4* overexpressing plants accumulate 26% more Pi in brown rice, so OsPHT1;4 may be involved in Pi remobilization from flag leaves and its translocation to the panicle. However, it is worth noting that *OsPHT1;4* expression after pollination is also enhanced in the roots. Thus, higher concentration of Pi in the transgenic rice grains may be a sum of increasing Pi uptake, translocation and remobilization, especially considering that in field conditions this rice accumulates about two times more Pi in the straw, while RNAi lines are characterized by fewer tillers and roots (Ye et al., 2015). Detailed investigation of OsPHT1;6 and OsPHT1;8 revealed their roles in Pi remobilization from senescing to young leaves and rice grains. *OsPTH1;6* overexpression is correlated with higher total P concentration in the shoot tissue and increasing in shoot biomass. Plants have also significantly higher P content in young leaves, compared to old leaves, that in WT plants (Zhang F. et al., 2014). Analogically, selective knockdown of *OsPHT1;8* only in the rice shoot caused increase in Pi and total P concentration in old leaves by 50–250%, altered expression of phosphate starvation induced genes in young and old leaves, and reduced total P content in both embryo and endosperm (Li et al., 2015). In contrast, in P-replete conditions, overexpression of *OsPHT1,1* resulted in increased Pi concentration in xylem sap and young leaves, but not in lower Pi concentration in old leaves. This clearly indicates that these changes are caused by enhanced Pi uptake and/or allocation to aerial plant organs, but not by increased remobilization of phosphate from senescent leaf blades (Sun et al., 2012).

## Phosphate Movement in Reproductive Tissues

During flowering and seed formation, transport of Pi from source organs to reproductive organs occurs. It is well-documented that Pi availability in the soil and its concentration in plant tissues have a strong impact on plant reproductive success (Lau and Stephenson, 1994; Zhang Z. et al., 2014). In the developing seeds, Pi and inositol are used for synthesis of phytic acid (myo-inositol-hexakisphosphate). Phytic acid creates salts with potassium, calcium, magnesium and iron, commonly named phytate, which is the main form of phosphate storage in the endosperm and embryo. During germination, due to activity of phytases, phytate is broken down, and phosphate, inositol and cations are released and are using for seedling growth and development (Azeke et al., 2011; Li et al., 2015). Moreover, Pi is not only transported to female organs and, after pollination, to the newly formed embryo, but is also stored in the pollen. It was shown that pollen grains from plants growing in Pi-rich soil have a higher Pi concentration and pollen grain volume. Because P stored in the pollen is used for example for phospholipid synthesis, which occurs rapidly during pollen tube growth, pollen from plants growing in high Pi concentration sired more seeds when competing for the same ovule with pollen from Pi-stressed plants (Lau and Stephenson, 1994).

Relatively little is known about phosphate transport into and out of *Arabidopsis* flowers and seeds. Although, all *PHO1* homologs, except *PHO1;H2*, were reported to be expressed in some parts of flowers or germinating pollen grains (in the petals, sepals or filament vasculature, receptacle, stigma apex, anther connectives, pollen grains or pollen tube), their role in flower development is enigmatic (Wang et al., 2004). In flower buds, among *AtPHT1* representatives, only activity of the *AtPHT1;5* promoter was detected (Mudge et al., 2002). However, weak *AtPHT1;9* expression in flowers was demonstrated by RT-PCR analysis, but unfortunately the authors did not specify in which flower part or in which flower developmental stage (Remy et al., 2012). During development of gametophytes Pi is transported to the growing ovule and microspores. Although no *AtPHT1* gene expression in *Arabidopsis* pistils was detected, in mature pollen expression of *AtPHT1;6* and *AtPHT1;7* was reported. *AtPHT1;6* promoter activity was also noted in anthers, especially in the tapetum (Mudge et al., 2002), the cells supplying nutrients to developing microspores as well as being a source of lipids composing the pollen coat (Furness and Rudall, 2001). For Pi transport to *Arabidopsis* seeds, AtPHT1;9 (Remy et al., 2012) and probably AtPHT1;5 (Nagarajan et al., 2011) are responsible. Quantitative trait locus (QTL) analysis identified *AtPHT1;9* as a marker of Pi concentration in the seeds, although the *AtPHT1;9* transcript level in the *Arabidopsis* siliques was low and no germination defects were detected in the *atpht1;9* mutant (Remy et al., 2012). Overexpression of *AtPHT1;5* leads to twofold higher Pi concentration in the siliques, but this effect may arise from enhanced Pi export from old leaves, not from the direct influence of AtPHT1;5 on Pi transfer to the seeds (Nagarajan et al., 2011). Finally, in the fading flowers (in the senescing anther filaments and sepals respectively) *AtPHT1;4* and *AtPHT1;5* expression occurs. *AtPHT1;4* is also specifically expressed in the silique abscising zone. This probably reflects the ability of *A. thaliana* to re-mobilize Pi out of aging flowers and fruits to support growth of other plant organs (Karthikeyan et al., 2002; Mudge et al., 2002).

More investigations have been focused on OsPHT1 transporters in rice grains. Rice is the staple food for around 50% of the world’s population (Zhang F. et al., 2014), so over-expression or down-regulation of particular *OsPHT1* in this crop may have a practical, measurable impact. Based on high expression in the embryo, three genes have emerged as important for development of rice seeds – *OsPHT1;1*, *OsPHT1;4*, and *OsPHT1;8* (Zhang et al., 2015) – and involvement of two of them – *OsPHT1;4* (Zhang et al., 2015) and *OsPHT1;8* (Jia et al., 2011; Li et al., 2015) – in this process was confirmed. *OsPHT1;4* expression in the embryo was around 10 times higher than in the endosperm or panicle axis. Knockdown and knockout of *OsPHT1;4* caused a 21–24% reduction of the total P amount in the embryo, but not in the endosperm, a 22–32% decrease in phytic acid concentration in the seeds as well as downregulation of genes involved in phytic acid synthesis and lower embryo size. In contrast, *OsPHT1;4* overexpressing lines have higher total P content in the embryo and endosperm, higher phytic acid concentration and higher expression of phytic acid synthesis enzymes than wild type. But, what is more important, altered expression of *OsPHT1;4* affects panicle performance, grain filling and seed germination of transgenic *O. sativa*. The panicles of knockout and RNAi lines are less robust and have 50–58% lower seed-setting rates than WT. Grain yield per plant and 1000-grain weight in knockout and knockdown rice lines also decreased. In consequence of mutation or downregulation of *OsPHT1;4*, germination of seeds is retarded: emergence of the radical and plumule occurs later after imbibition, and the shoot and root of young seedling are shorter than in WT plants. In contrast, *OsPHT1;4* overexpressors have higher 1000-grain weight and percentage germination rate (Zhang et al., 2015). Similarly, downregulation or mutation of *OsPHT1;8* results in 30% higher total P content in the panicle axis, 30% lower total P content in unfilled grain hulls and a decreased seed-setting rate (Jia et al., 2011), while selective attenuation of *OsPHT1;8* in seed endosperm leads to a 40–50% reduction of total P content in the embryo but not in the endosperm, compared with WT (Li et al., 2015). This implies OsPHT1;8 participation in Pi transfer from the panicle axis to the seeds (Jia et al., 2011) and from the endosperm to the embryo (Li et al., 2015). Although some similarities between *OsPHT1;4* and *OsPHT1;8* functioning exist, we can speculate that they play a different role in rice grain development. In *ospht1;4* and *OsPHT1;4* RNAi plants transcript levels of *OsPHT1;1* and *OsPHT1;8* are significantly increased, but they do not compensate disruption or suppression of *OsPHT1;4* (Zhang et al., 2015). Moreover, the microarray expression profile of all 26 *PHT* rice genes in 27 tissues covering the plant lifecycle in three rice cultivars did not show higher expression of *OsPHT1;1*, *OsPHT1;4*, or *OsPHT1;8* in germinating rice seeds but 72 h of the imbibition stage specific and high expression of another gene, *OsPHT1;12*, were detected (Liu et al., 2011). *OsPHT1;12* together with *OsPHT1;7*, are also strongly expressed in the anthers (Gu et al., 2016).

## Intracellular Pi Transporters

### Pi Transport Across the Vacuole Membrane

The translocation of Pi to subcellular organelles is crucial for metabolic regulation and homeostasis of Pi in the plant cell. The vacuole plays a central role in Pi sequestration, and it is the major Pi storage compartment (Raghothama, 1999). Under Pi-sufficient conditions, to prevent toxicity of cytoplasm, Pi excess is stored in the vacuole, whereas under Pi deficiency inorganic phosphate is exported from the vacuole to the cytosol (Shane et al., 2004). However, very little is known, compared to other organelles, about protein exchangers that are required for movement of Pi in and out of the vacuole (Bucher and Fabiańska, 2016). Two independent research groups, using different technics for the functional characterization of vacuolar Pi transporters, made an important discovery. Liu et al. (2015) using patch-clamping methods, identified in *A. thaliana* an ortholog of OsSPX-MFS1, namely VPT1, that operates as an influx transporter in the tonoplast (**Figure 3**). A number of genetic studies on the *vpt1* and electrophysiological approaches suggested that VPT1 might act as an anion channel in the tonoplast (Liu et al., 2015). The VPT1 was also named PHT5;1 in the article by Liu T. et al. (2016). They determined using ^31^P- MRS (magnetic resonance spectroscopy) the translocation of Pi across the tonoplast in wild type plants (Liu T. et al., 2016). The knock-out mutants of PHT5;1 exhibited toxic concentration of Pi in cytoplasm. Furthermore, the analyses of *Arabidopsis* mutants and overexpression lines suggested that two other proteins – PHT5;2 and PHT5;3- also participated in vacuolar Pi sequestration (Liu T. et al., 2016). Previously, vacuolar transporters have been studied only in yeast and rice. In *O. sativa* three proteins belonging to the SPX-MFS family: OsSPX-MFS1, OsSPX-MFS2, OsSPX-MFS3, have been identified as involved in Pi transport across the vacuolar membrane (Wang et al., 2015). The SPX-MFS (Major Facilitator Superfamily) proteins contain 10–11 transmembrane domains and function as transport carriers (uniporters, symporters, or antiporters) for different substrates, i.e., ions and organic compounds (Secco et al., 2012). It is suggested that these proteins are key players in Pi signaling, transport and remobilization in leaves (Lin et al., 2010). Overexpression of *OsSPX-MFS1* in yeast cells and complementation of *pht5;1* with *OsSPX-MFS1* indicated that OsSPX-MFX1 acts as a Pi channel (Liu F. et al., 2016). Whereas OsPSX-MFS3 is proposed as a vacuolar efflux Pi:H^+^ symporter (Wang et al., 2015; **Figure 3**).

### Pi Transport Across the Mitochondrial Membrane

The mitochondrial phosphate transporters (MPTs) are responsible for transporting Pi into the mitochondrial matrix, where Pi is utilized for oxidative phosphorylation of ADP to ATP by ATP synthase (Monné et al., 2015). MPTs act as Pi:H^+^ symporters or Pi/OH^-^ antiporters between mitochondria and the cytosol (Stappen and Krämer, 1994). In *A. thaliana* phosphate transport across the mitochondrial membrane is mediated by PHT3 proteins (**Figure 1**) belonging to the mitochondrial carrier family (MCF), together with ADP/ATP carriers (Haferkamp and Schmitz-Esser, 2012; Lorenz et al., 2015). PHT3s in *A. thaliana* are encoded by three genes, named *AtPHT3;1*, *AtPHT3;2*, and *AtPHT3;3* (Poirier and Bucher, 2002). Most are expressed in multiple cell layers in stems, leaves and flowers (Zhu et al., 2012). It was reported that *AtPHT3;1* and *AtPHT3;2* are strongly upregulated by salt stress, and overexpression of these genes leads to increased sensitivity to salt stress in *Arabidopsis* seedlings (Zhu et al., 2012).

Some studies have reported that cDNA encoding MPT proteins was isolated from other plants, i.e., soybean, maize, and *Lotus japonicas* (Takabatake et al., 1999; Nakamori et al., 2002). So far, only one report, by Liu et al. (2011) has described mitochondrial PHT family genes in the rice genome. Using the microarray method, the expression of all 26 genes of the *PHT* family was characterized. Moreover, the phylogenetic tree of PHT was generated, based on full-length amino acid sequences from *Arabidopsis* and *O. sativa*. One of the four clusters, namely cluster III, contains nine members: three from *Arabidopsis* (AtPHT3;1 – AtPHT3;3) and six from rice (OsPT15, 16, 17, 18, 19, 20; Liu et al., 2011). However, the same phylogenetic grouping does not mean the same subcellular localization, because none of these six proteins (OsPT15-20) were found in mitochondria, like the mentioned PHT3 members from *Arabidopsis*. OsPT15, 17, 18, 19 are located in peroxisomes, OsPT16 on the endoplasmic reticulum and OsPT17 on the plasma membrane (Liu et al., 2011).

### Pi Transport Across the Golgi System

The Golgi apparatus plays a fundamental role in intracellular trafficking, protein glycosylation, and non-cellulosic polysaccharide synthesis in plant cells. The wall polysaccharides are synthesized from nucleotide sugar substrates, which are transported into the Golgi by nucleotide sugar transporters (NSTs) and here nucleoside diphosphates are hydrolyzed to nucleoside monophosphates and Pi (Driouich et al., 2012). It has been shown that a specific transporter exports Pi out of the Golgi. Subcellular localization using GFP fusion confirmed that only one member of the PHT4 family of intracellular Pi transporters is targeted to the Golgi apparatus in *Arabidopsis* (**Figure 3**). AtPHT4;6 mediates the transport of Pi from the Golgi toward the cytosol (Guo et al., 2008a). The role of AtPHT4;6 as a Pi exporting protein was supported by identification and characterization of the at*pht4;6* mutant, which is hypersensitive to salt stress, especially in plants growing under Pi starvation (Cubero et al., 2009). Disruption of the At*PHT4;6* gene results also in strong growth inhibition and alteration of cell wall composition with reduced levels of rhamnose and mannose and increased levels of fructose and arabinose (Hassler et al., 2012). Two homologs of AtPHT4;6 have been found in the rice secretory system (Guo et al., 2008b). However, our knowledge on phosphate transport across endomembranes in rice is still limited.

### Phosphate Transport in Plastids

**FIGURE 4:**
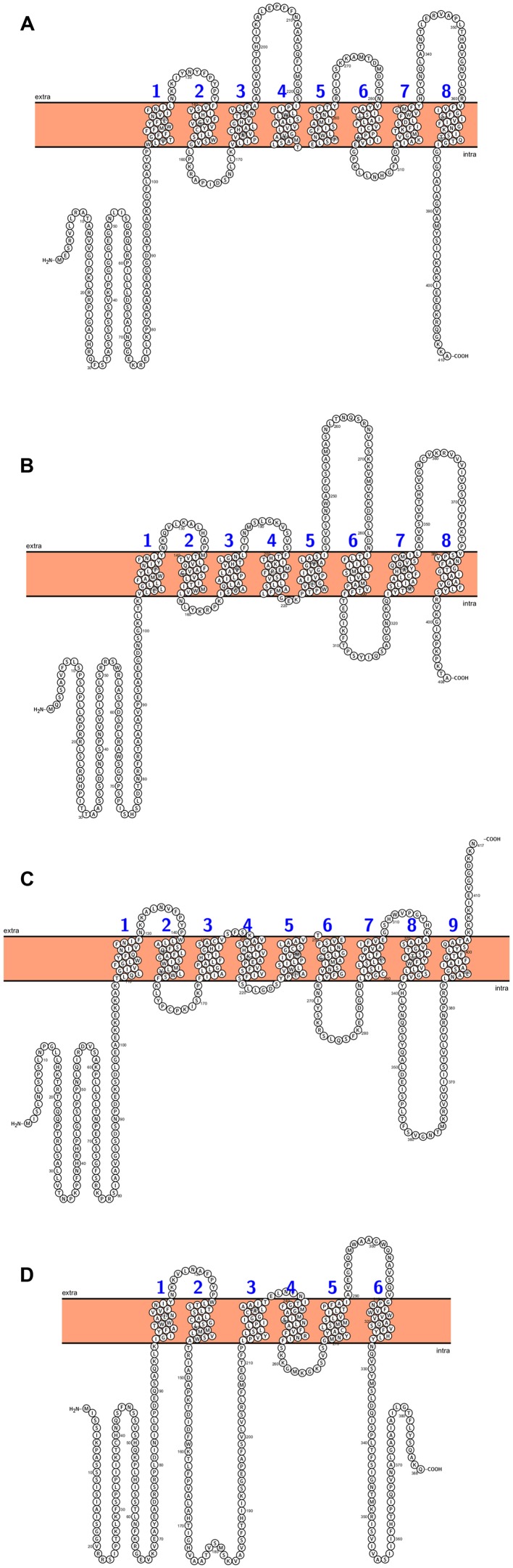
***Arabidopsis* chloroplast’s phosphate translocators predicted topology: (A) TPT (triose-phosphate/phosphate translocator), (B) PPT1 (phosphoenolpyruvate/phosphate translocator), (C) XPT (xylulose-5-phosphate/phosphate translocator), (D) GPT2 (glucose-6-phosphate/phosphate translocator).** All figures were performed in Protter (Protter: interactive protein feature visualization and integration with experimental proteomic data. Omasits et al., 2014), protein sequences come from UniProt database.

In plastids three classes of Pi transporters have been characterized: PHT2, PHT4 and Pi translocators (**Figures 1**, **3**, and **4**; Poirier and Bucher, 2002; Rausch and Bucher, 2002). AtPHT2;1 is currently the only transporter of the PHT2 family identified in *Arabidopsis* (**Figure 1**). The cDNA encodes a 12-transmembrane protein with high homology to fungal and mammalian Na^+^/Pi transporters (Daram et al., 1999). The *AtPHT2;1* gene is predominantly expressed in green tissue and AtPHT2-GFP fusion protein indicates that AtPHT2;1 is located in the chloroplast inner envelope membrane (Versaw and Harrison, 2002). Mutant *atpht2;1* reveals reduced transport into the chloroplast and contains 20 times less Pi compared to the wild type (Versaw and Harrison, 2002). Similar to *Arabidopsis AtPHT2;1* the rice ortholog *OsPHT2;1* is expressed mainly in leaves and is induced by phosphate deficiency and light (Shi et al., 2013).

The functional analysis of the PHT4 family (**Figure 1**) and subcellular localization in *Arabidopsis* of all 5 members AtPHT4;1 – AtPHT4;5 were described in detail by Guo et al. (2008a). These proteins share similarities with mammalian SLC17/type I transporters that are involved in the transport of Pi and chloride (Reimer and Edwards, 2004). AtPHT4;1, AtPHT4;2, AtPHT4;4, and AtPHT4;5 have been visualized in plastids using protein GFP fusion. More advanced analyses of purified plastids and GUS activity revealed that AtPHT4;1 and AtPHT4;4 are localized in leaf chloroplasts, AtPHT4;2 in root plastids, and AtPHT4;3 and AtPHT4;5 in shoot plastids (Guo et al., 2008a). The AtPHT4;1, also known as the anion transporter ANTR1, has been characterized as a H^+^-dependent high affinity Pi transporter when expressed in yeast (Guo et al., 2008b) and Na^+^-dependent transporter using *Escherichia coli* system (Pavón et al., 2008). It was also documented that AtPHT4;2 (Irigoyen et al., 2011) and AtPHT4;4 (Finazzi et al., 2015) mediate H^+^ or Na^+^ dependent Pi symport. Mutation of the *AtPHT4;1* gene (*atpht4;1*) leads to a dwarf phenotype with smaller leaf rosette and reduced biomass compared to WT plants (Karlsson et al., 2015). Due to lower level of Pi in *pht4;1* mutant, the activity of ATP-synthase is inhibited and this in turn, leads to decrease level of ATP and reduction in accumulation of soluble sugars, which could limit plant growth (Karlsson et al., 2015). Moreover, mutant *pht4;1* was more susceptible to infection with virulent bacterium *Pseudomonas syringae*, but salicylic acid induced pathogen resistance in this mutant (Wang et al., 2011). Therefore, it was proposed that AtPHT4;1 is required to control the immune response to pathogen infection and its expression is dependent on circadian clock protein CCA1 (Wang et al., 2011; Wang G. et al., 2014). Furthermore, Miyaji and co-workers speculated that AtPHT4;1 may transport ascorbate into the lumen to overcome photoinhibition caused by strong light (Miyaji et al., 2015). However, evaluation of ascorbate content at high light conditions conducted independently by Karlsson et al. (2015) did not reveal any differences between WT and the *atpht4.1* mutant line. Thus, the hypothesis that AtPHT4;1 transports ascorbate into the lumen has not been confirmed. According to obtained data from proteoliposome assay, other protein, namely, AtPHT4;4 might transport ascorbate (Miyaji et al., 2015). *Atpht4;4* mutant exhibited reduced level of ascorbate (about 30%) in leaves and defect in the xanthophyll cycle. *AtPHT4;4* is expressed in chloroplast and its level increased under light exposure (Miyaji et al., 2015). It has been documented that AtPHT4;2 contributes to Pi transport in root plastids and expression of the *AtPHT4;2* gene is restricted to roots and floral tissue (Irigoyen et al., 2011). The *atpht4;2* mutant surprisingly exhibited a larger rosette size, caused by increased cell proliferation in the leaf, as well as reduced starch biosynthesis in shoots as an inhibitory effect of Pi excess on ADP-glucose pyrophosphorylase, the key enzyme in starch synthesis (Irigoyen et al., 2011). Based on TIGR rice genome database seven protein sequences of PHT4 was found in rice and they share about 70–80% similarity to *Arabidopsis* orthologs (PHT4;1, 4;2, 4;3; 4;4, 4;5 and two homologs of PHT4;6; Guo et al., 2008b).

Several transporters localized in the inner envelope membrane connect the metabolism processes between chloroplasts, mitochondria, and surrounding cytosol. There are 4 plastid translocators: triose-phosphate/phosphate translocator (TPT), exporting the photosynthetically fixed carbon in a form of triose-phosphates, the phosphoenolpyruvate/phosphate translocator (PPT) that delivers the phosphoenolpyruvate from the cytosol, the pentose xylulose-5-phosphate/phosphate translocator (XPT), and the glucose-6-phosphate/phosphate translocator which mediates import of carbon skeletons to non-photosynthetic plastids (Weber, 2004; Flügge et al., 2011). Phylogenetic analyses showed that plastid phosphate translocators originated from an red algal ancestor and their homologs in other plants (cauliflower, tobacco, pea, spinach, potato, maize) were founded. (Kammerer et al., 1998; Weber et al., 2006; Weber and Linka, 2011). The *Arabidopsis* genome contains a single copy for *TPT* and *XPT* genes, and two genes encoding PPT and GPT, respectively (**Figure 4**). *TPT* genes are expressed primarily in photosynthetic tissue. *GPT* is expressed only in non-photosynthetic plastids, whereas *PPT* are expressed in both photosynthetic and non- photosynthetic tissues (Knappe et al., 2003). The *cue1* (chlorophyll a/b binding protein CAB gene underexpressed 1) mutant is defective in PPT translocator and was first time described by Li et al. (1995). Mutation at this gene resulted in reticulate leaf phenotype and reduction of chlorophyll and carotenoids content (Li et al., 1995; Streatfield et al., 1999). Mutants that lack the activity of TPT (*tpt1*) show similar phenotype to that of WT plants without any changes in leaves size or in fresh weight (Schneider et al., 2002). Disruption of GPT1 resulted in several defects, especially during pollen development (pollen with reduced storage lipids) and lower concentration of starch in leaves (Niewiadomski et al., 2005). Loss of GPT2 has no effect on plant growth and development, whereas mutation of *GPT1* is lethal (Niewiadomski et al., 2005). The genes encoding the putative plastid translocators in rice were identified and they were named after *Arabidopsis* homologs. Thus, two functional rice *TPT* genes (*OsTPT1,OsTPT2*), four *GPT* (*OsGPT1*, *OsGPT2-1*, *OsGPT2-2*, *OsGPT2-3*), and four *PPT* genes (*OsPPT1, OsPPT2, OsPPT3, OsPPT4*) were found in rice genome and their expression patterns in various organs were examined by Toyota et al. (2006). The *OsTPT* and *OsPPT* were expressed predominantly in leaves, whereas the expression of the *OsGPT1*, *OsGPT2-1* and *OsGPT 2-2* was much higher in seed compared to the photosynthetic tissues. None of rice xylolose-5-phosphate genes and proteins (XPT) have been characterized so far (Toyota et al., 2006).

### Summary

In summary, many transporter proteins and their respective genes have been identified and characterized, but the physiological role of Pi transporters in response to Pi starvation is still very limited. Understanding of the molecular mechanism involved in the regulation of phosphate transporters is very important, due to the constantly lower availability of phosphorus reserves in nature, and will facilitate the development of more efficient Pi-utilizing plants.

## Author Contributions

All authors listed, have made substantial, direct and intellectual contribution to the work, and approved it for publication.

## Conflict of Interest Statement

The authors declare that the research was conducted in the absence of any commercial or financial relationships that could be construed as a potential conflict of interest.
