# Supplementary material for: Phosphate Uptake and Allocation – A Closer Look at Arabidopsis thaliana L. and Oryza sativa L
Source: Front Plant Sci. 2016 Aug 15;7:1198. doi: 10.3389/fpls.2016.01198 (PMC4983557; doi:10.3389/fpls.2016.01198)
Supplement: Supplementary file 1 [file Table_1.DOCX]

Supplementary Material

**Phosphate uptake and allocation – a closer look at *Arabidopsis thaliana* L. and *Oryza sativa* L.**

Ewa Młodzińska*, Magdalena Zboińska

*** Correspondence:** ewa.mlodzinska@uwr.edu.pl

**Table 1.** **Phosphate transporters and their genes in plant species**

| Plant species^a^ | Gene^b^ | Transcript location | Affinity^c^ | Mycorrhiza^d^ | Commments | References^e^ |
| --- | --- | --- | --- | --- | --- | --- |
| *Astragalus sinicus/* Chinese milkvetch | *AsPHT1;1* | Roots | High (Km = 31,12 ± 5,87 µM) | ↑AM | Expressed only in mycorrhizal roots. Plants overexpressing *AsPHT1;1* have higher mycorrhization levels. *AtPHT1;1* down-regulation led to degenerating or dead arbuscule phenotypes but *AsPHT1;1* is not required for symbiotic Pi uptake. | Xie et al., 2013 |
|  | *AsPHT1;2* | Roots | Low (based on spot-tests results) | ↓AM | Expressed in epidermis and root tip. |  |
|  | *AsPHT1;3* | Roots | Low (Km = 128,7 ± 60,17 µM) | ↑AM | Expressed in the root cortex cells. |  |
|  | *AsPHT1;4* | Roots | Low (based on spot-tests results) | ↑AM | Expressed only in mycorrhizal roots. Required for symbiotic Pi uptake. *AtPHT1;4* knockdown  leads to degenerated or dead arbuscules  phenotypes. |  |
|  | *AsPHT1;5* | Roots | High (Km = 46,75 ±19.54 µM) | ↓AM | Expressed in central cylinder and root tip. |  |
|  | *AsPHT1;6* | Roots | - | - | - |  |
| *Brachypodium*  *distachyon/* purple false brome | *BdPHT1;1* | Roots, shoots | - | - | - | Hong et al., 2012 |
|  | *BdPHT1;2* | - | - | - | Not-detectable in roots and shoots. |  |
|  | *BdPHT1;3* | Roots | - | ↑AM | Specifically induced in AM roots. |  |
|  | *BdPHT1;4* | Roots | - | AM-no influence |  |  |
|  | *BdPHT1;5* | - | - | - | Not-detectable in roots and shoots. |  |
|  | *BdPHT1;6* | - | - | - | Not-detectable in roots and shoots. |  |
|  | *BdPHT1;7* | Roots, shoots | - | ↑AM (roots), ↓AM (shoots) | Specifically induced in roots by AM fungi. Weakly expressed in shoots, down-regulated upon AM formation. |  |
|  | *BdPHT1;8* | Roots, shoots | - | ↓AM (shoots) | Not expressed in shoots of AM fungi colonized plant. |  |
|  | *BdPHT1;9* | Root, shoots | - | ↓AM (shoots) | Down-regulated in shoots upon AM formation. |  |
|  | *BdPHT1;10* | Shoots | - | ↓AM (shoots) | Weakly expressed in shoots of non-mycorrhizal plants. |  |
|  | *BdPHT1;11* | Roots, shoots | - | AM-no influence | Strongly expressed in roots and shoots. |  |
|  | *BdPHT1;12* | Roots, shoots | - | ↑AM (roots), ↓AM (shoots) | Specifically induced in AM roots. Weakly expressed in shoots of non-mycorrhizal plants. |  |
|  | *BdPHT1;13* | Roots | - | ↑AM | Specifically induced in AM roots. |  |
| *Brassica napus*/rapeseed | *BnPHT1;4* | Roots, hypocotyl, cotyledons, stems, leaves, flowers | - | - | Organ-specific expression was prepared on plants growing at high Pi (1 mM). In these conditions *BnPHT1;4* is highly expressed in stems and flowers. In roots *BnPHT1;4* is strongly induced by Pi deficiency (this induction is dependent on the presence of sugars). The gene overexpression in transgenic *Arabidopsis* causes changes in root system architecture. | Ren et al., 2014 |
| *Capsicum frutescens/*chilli pepper | *CfPHT1;1* | Roots, leaves | - | ↑AM (weak) | Up-regulated by Pi deficiency in roots and leaves. | Chen et al., 2007 |
|  | *CfPHT1;2* | Roots | - | ↑AM | In non-mycorrhizal roots strongly up-regulated under Pi-deficiency. During symbiosis expressed also at high Pi concentration. |  |
|  | *CfPHT1;3* | Roots, leaves | - | ↑AM | Expression is strongly enhanced by AM formation but only in P-deprived roots. Weakly expressed in leaves. |  |
|  | *CfPHT1;4* | Roots | - | ↑AM | Induced by AM formation, especially in Pi-deprived roots. |  |
|  | *CfPHT1;5* | Roots | - | ↑AM | Induced by AM formation only in Pi-deprived roots. |  |
|  | *CfPHT2;1* | Leaves | - | ↓AM | Not expressed in leaves. Strongly up-regulated by Pi-deficiency but not in AM plants. Not detected in roots. |  |
| *Catharanthus roseus*/ periwinkle | *CrPHT1;1* | Roots, stems, but not leaves | - | - | Named also PIT1. | Kai et al., 1997 |
| *Chrysanthemum morifolium/* Florist's daisy | *CmPHT1* | Roots, stems, but not leaves | High (Km = 35,2 μM) | - | Strongly expressed in root tissues and weakly in stems. In roots, but not stems, expression is induced by low Pi concentration. *CmPHT1* overexpressing transgenic plants take up more phosphate, grow taller, produce greater volume of roots and accumulate  more biomass than not-transformed plants, under both Pi-sufficient and Pi-deficient conditions. | Liu et al., 2014 |
| *Eucalyptus camaldulensis/* river red gum | *EcPHT1;1 – EbPHT1;5* | - | - | - | Putative genes extracted from the NCBI GeneBank database. | Loth-Peredea et al., 2011 |
| *Eucalyptus grandi /* flooded gum | *EgPHT1;1-*  *EgPHT1;16* | - | - | - | Putative *PHT1* genes extracted from the Phytozome database. | Kariman et al., 2016 |
| *Eucalyptus marginata*/ jarrah | *EmPHT1;1* | Roots | - | ↓ECM, AM – no influence, non-colonizing symbiosis – no influence | Expression enhanced by Pi, Phi (phosphite) and AsV (arsenate) after 1 day of treatment. AM results obtained after colonization by *Scutellospora calospora*. ECM (ectomycorrhiza) results obtained after colonization by *Scleroderma* sp. which sometimes  establishes non-colonizing symbiotic association and sometimes ECM symbiosis, probably dependently on temperature. Other results from non-colonizing symbiosis are obtained for *Austroboletus occidentalis,* which always forms non-colonizing symbiosis. | Kariman et al., 2014; 2016 |
|  | *EmPHT1;2* | Roots | - | ↓ECM; AM – no influence, non-colonizing symbiosis – no influence | Expression enhanced by Pi and not changed by Phi and AsV after 1 day of treatment. Fungus species used for experiments: *Scutellospora calospora*. *Scleroderma* sp. and *Austroboletus occidentalis*. |  |
|  | *EmPHT1;3* | Roots | - | AM and non-colonizing symbiosis – no influence | Expression enhanced by Pi, Phi and AsV after 1 day of treatment. AM fungus - *Scutellospora calospora,*  non-colonizing symbiotic fungi- *Scleroderma* sp. and *Austroboletus occidentalis* |  |
|  | *EmPHT1;4* | Roots | - | AM and non-colonizing symbiosis – no influence | Expression enhanced by Pi, Phi and AsV after 1 day of treatment. AM fungus - *Scutellospora calospora,*  non-colonizing symbiotic fungi- *Scleroderma* sp. and *Austroboletus occidentalis* |  |
|  | *EmPHT1;5* | Roots | - | ECM -no influence, AM – no influence, non-colonizing symbiosis – no influence | No expression changes after 1 day of treatment by Pi, Phi or AsV. Fungus species used for experiments: *Scutellospora calospora*. *Scleroderma* sp. and *Austroboletus occidentalis*. |  |
| *Glycine max/* soybean | *GmPHT1;1* | Roots, hypocotyls, epicotyls,  stems,  cotyledons unifoliolate  leaves, the first trifoliolate leaves, the second trifoliolate leaves, the third trifoliolate leaves, the fourth trifoliolate leaves flowers, pods. | Low (Km =  105,30 $\pm$ 20,00 µM or 68,94 µM  or 6,65 mM; dependently on studies) | ↓AM/ AM-no influence (depends on study) | Strongly expressed in roots as well as in cotyledons and in unifoliolate leaves during flowering stage. Weakly expressed in pods/flowers (different results were obtained in different studies). Expressed under high and low Pi concentration. In the response to the low Pi stress *GmPHT1;1* expression in stems is down-regulated but in the roots and unifoliolate leaves is weakly up-regulated. Potassium, nitrogen and iron deficient lead to *GmPHT1;1* up-regulation in leaves. Low nitrogen stress causes also gene up-regulation in roots. | Wu et al., 2011; Qin et al., 2012a, 2012b; Tamura et al., 2012; Fan et al., 2013; Inoue et al., 2014 |
|  | *GmPHT1;2* | Roots, hypocotyls, epicotyls,  stems,  cotyledons unifoliolate  leaves, the first trifoliolate leaves, the second trifoliolate leaves, the third trifoliolate leaves, the fourth trifoliolate leaves flowers, pods | High (Km = 44 $\pm$ 12,9 µM) or low  (Km = 167,4 µM) -dependently on studies | ↓AM | Dependently on studies strong expression in mature pods or weak expression in pods and flowers but strong in roots were observed. Expressed under high and low Pi concentration. In the response to the low Pi *GmPHT1;2* expression in stems is strongly down-regulated but in the roots and unifoliolate leaves is up-regulated (weakly in roots, stronger in leaves). *GmPHT1;2* expression in roots is also strongly up-regulated by nitrogen and potassium deficiency. |  |
|  | *GmPHT1;3* | Roots, hypocotyls, epicotyls,  stems,  cotyledons unifoliolate  leaves, the first trifoliolate leaves, the second trifoliolate leaves, the third trifoliolate leaves, the fourth trifoliolate leaves flowers, pods | Low (Km = 65,30 ± 13,30 µM) | ↓AM | Weakly expressed in all tissues, the weakest in epicotyls and cotyledons. In the response to the low Pi stress *GmPHT1;3* expression in stems and unifoliolate leaves is down-regulated but in roots is up-regulated. In roots up-regulated by potassium deficiency. |  |
|  | *GmPHT1;4* | Rootshypocotyls, epicotyls,  stems,  cotyledons unifoliolate  leaves, the first trifoliolate leaves, the second trifoliolate leaves, the third trifoliolate leaves, the fourth trifoliolate leaves flowers, pods | High (Km = 25,70 $\pm$1,63 µM) | ↓AM /AM-no influence (depends on study) | The higher *GmPHT1;4* mRNA level was detected in unifoliate leaves during flowering stage. In other study strongly expressed in flowers. Up-regulated in roots (weakly), stems (around 6-times) and unifoliolate leaves (weakly) during Pi deficiency stress. In leaves up-regulated by nitrogen deficiency, in roots up-regulated by low potassium stress. *GmPHT1;4* is expressed in the junction area between roots and nodules as well as in the nodule vascular bundles. This transporter is responsible for Pi transfer from the root vascular system to nodules and regulates nodules formation. |  |
|  | *GmPHT1;5* | Roots, hypocotyls, epicotyls,  stems,  cotyledons unifoliolate  leaves, the first trifoliolate leaves, the second trifoliolate leaves, the third trifoliolate leaves, the fourth trifoliolate leaves flowers, pods. | High  (Km = 32,30 $\pm$1,60 µM or  25 µM)  or low (Km =  243,9 µM) - dependently on studies | ↓AM | Weakly expressed in epicotyls/flowers (dependently on study). The strongest expression was detected in the first trifoliolate leaves. In the response to the low Pi stress *GmPHT1;5* expression in stems is strongly down-regulated (more than 5 times). In roots and leaves up-regulated by phosphate, nitrogen and iron deficientcy stress. |  |
|  | *GmPHT1;6* | Roots, hypocotyls, epicotyls,  stems,  cotyledons unifoliolate  leaves, the first trifoliolate leaves, the second trifoliolate leaves, the third trifoliolate leaves, the fourth trifoliolate leaves flowers, pods. | Low (Km = 153 $\pm$ 44 µM or  373,4 µM - dependently on studies) | - | Strongly expressed in leaves and flowers. In other organs very low expression level. In leaves *GmPHT1;6* expression is up-regulated by nitrogen deficiency. In the response to the low Pi stress Fan et al. (2013) showed strongly down-regulation of *GmPHT1;*6 expression in stems (more than 6 times) but up-regulation in the roots and unifoliolate leaves but in Qin et al. (2012) study gene was up-regulated by low Pi in roots, young leaves, stems and flowers. |  |
|  | *GmPHT1;7* | Roots, stems,  cotyledons unifoliolate  leaves, the first trifoliolate leaves, the second trifoliolate leaves, the third trifoliolate leaves, the fourth trifoliolate leaves flowers, pods. | Low (Km = 79,00 $\pm$8,30 µM or 145,2 µM - dependently on studies) | ↓AM/ AM-no influence (depends on study) | The highest mRNA level was detected in roots during flowering stage. Not detectable in epicotyls and hypocotyls, weakly expressed in mature pods. Up-regulated in roots (around 8-times), stems (around 5-times) and unifoliolate leaves (around 2-times) during Pi deficiency stress. In leaves up-regulated by nitrogen deficiency. |  |
|  | *GmPHT1;8* | Roots, hypocotyls, epicotyls,  stems,  cotyledons unifoliolate  leaves, the first trifoliolate leaves, the second trifoliolate leaves, the third trifoliolate leaves, the fourth trifoliolate leaves flowers, pods. | High (Km = 45,30 $\pm$14,53 µM) | ↓AM /AM-no influence (depends on study) | Co-localized with ER markers, not detectable in plasma membrane. The highest expression in cotyledons, high in shoots, roots, hypocotyls, epicotyls and in the fourth trifoliolate leaves. Expression in other types of leaves is very weak. In pods expression decreased during maturation. During low Pi stress weakly up-regulated in roots, weakly down-regulated in unifoliolate leaves and strongly (around 5-times) down-regulated in stems. Potassium, nitrogen and iron deficiency lead to *GmPHT1;8* up-regulation in roots. |  |
|  | *GmPHT1;9* | Roots, hypocotyls, epicotyls,  stems,  cotyledons unifoliolate  leaves, the first trifoliolate leaves, the second trifoliolate leaves, the third trifoliolate leaves, the fourth trifoliolate leaves flowers, pods. | High (Km = 30,00 ± 8,60) | ↓AM/ AM-no influence (depends on study) | The strongest expression was detected in seedling roots, unifoliolate leaves and the first trifoliolate leaves. Very low level of mRNA was detected in mature pods and epicotyls. In the response to low Pi stress up-regulated in roots and unifoliolate leaves and down-regulated in stems. *GmPHT1;9* expression in roots and leaves is also up-regulated by nitrogen, potassium and iron deficiency. |  |
|  | *GmPHT1;10* | Roots, hypocotyls, epicotyls,  stems,  cotyledons unifoliolate  leaves, the first trifoliolate leaves, the second trifoliolate leaves, the third trifoliolate leaves, the fourth trifoliolate leaves flowers, pods. | Low (Km = 116,30 $\pm$10,00 µM or 290 µM - dependently on studies) | - | Undetectable in seedlings roots, in other organs expression level is low. The highest level was noted in the fourth trifoliolate leaves. In the response to the low Pi stress *GmPHT1;10* expression in stems is down-regulated or up-regulated (results depend on studies) but in the roots, pods and unifoliolate leaves is up-regulated (in leaves more than 4 times). |  |
|  | *GmPHT1;11* | Roots, hypocotyls, epicotyls,  stems,  cotyledons unifoliolate  leaves, the first trifoliolate leaves, the second trifoliolate leaves, the third trifoliolate leaves, the fourth trifoliolate leaves flowers, pods. | Low (Km = 231,00 $\pm$ 15,60 µM) | ↑AM | Strongly expressed in roots during flowering stage. Expression in shoots, flowers and mature pods also higher than in other organs. In the response to low Pi stress up-regulated in roots and unifoliolate leaves and down-regulated in stems. In stem strongly (around 12-times) up-regulated by high Pi concentration in the medium. *GmPHT1;11* expression in roots is also up-regulated by potassium deficiency. *GmPHT1;11* is strongly up-regulated in senescing leaves (more precisely in the tips of vein endings at the late stage of Pi translocation from leaves to seeds). In roots detected in columella cells of root caps. |  |
|  | *GmPHT1;12* | Roots, hypocotyls, epicotyls,  stems,  cotyledons unifoliolate  leaves, the first trifoliolate leaves, the second trifoliolate leaves, the third trifoliolate leaves, the fourth trifoliolate leaves flowers, pods. | Low (Km = 88 $\pm$ 8,9 µM or 505,1 µM - dependently on studies) | ↑AM | Strongly expressed in shoots, in other organs expression is weak. In the response to the low Pi stress expression of *GmPHT1;12* is up-regulated in roots, stems and unifoliolate leaves. Potassium deficiency stress causes up-regulation of *GmPHT1;9* expression in the roots. |  |
|  | *GmPHT1;13* | Roots, hypocotyls, epicotyls,  stems,  cotyledons unifoliolate  leaves, the first trifoliolate leaves, the second trifoliolate leaves, the third trifoliolate leaves, the fourth trifoliolate leaves flowers, pods. | High (Km = 46,60 $\pm$ 5,60 µM) or low (Km = 363,6 µM) - dependently on studies | ↑AM | Weakly expressed in all organs, the strongest in roots and shoots during flowering stage. In the response to low Pi stress up-regulated in roots and unifoliolate leaves and down-regulated in stems. In leaves up-regulated by nitrogen deficiency, in roots by potassium deficiency. In seeds up-regulated by high Pi concentration. |  |
|  | *GmPHT1;14* | Roots | Low (Km = 67,30±15,60 µM) | ↓AM | Expression in seedling roots is around 3 times higher than in roots during flowering stage. *GmPHT1;14* mRNA level is up-regulated during Pi and potassium deficiency stress. |  |
|  | *GmPHT1;15* | Not detectable in any tested tissue. | - | - | Undetectable in all tested organs. |  |
| *Hakea prostrata/*harsh hakea | *HpPHT1;3/*  *HpPHT1;5* | Roots | - | - | The exact number of *PHT1* genes was described as ‘unknown’.  Genes were named basing on similarity to *Arabidopsis* transporters. | Lambers et al., 2013; Mirfakhraei, 2014 |
|  | *HpPHT1;4* | Roots, leaves | - | - |  |  |
|  | *HpPHT1;7* | - | - | - |  |  |
|  | *HpPHT1;8* | - | - | - |  |  |
|  | *HpPHT1;9* | - | - | - |  |  |
|  | *HpPHT2;1* | - | - | - | Genes were named basing on similarity to *Arabidopsis* transporters. |  |
|  | *HpPHT3;1* | - | - | - |  |  |
|  | *HpPHT3;2* | - | - | -- |  |  |
|  | *HpPHT3;3* | - | - | - |  |  |
|  | *Hp2000848* | Leaves | - | - | Genes which orthologs have not been found among *Arabidopsis PHT* genes. |  |
|  | *Hp2005506/*  *Hp2012485* | Roots, leaves | - | - |  |  |
|  | *Hp2018344* | Roots | - | - |  |  |
|  | *Hp2090243* | Leaves | - | - |  |  |
| *Hordeum vulgare/*barley | *HvPHT1;1* | Roots, leaves, ligue, auricle | High (Km = 9,06 ± 0.82 *μ*M or *K*m = 1.9 μM - dependently on studies) | ↓AM/↑AM (weak) (depend on study) | Able to transport Pi and arsenate, but not sulphate or nitrate. Up-regulated by low Pi concentration as well as by high potassium concentration. Sulphate or nitrogen deprivation reduce expression induced by low Pi. In primary roots expressed mainly in trichoblast cells and root apex. Down-regulated in older leaves and older parts of root. Probably responsible for Pi uptake from soil solution. | Smith et al., 1999; Rae et al., 2003; Schünmann et al., 2004a; 2004b; Glassop et al., 2005; Christophersen et al., 2009; Grace et al., 2009; Preuss et al., 2010; Huang et al., 2011; Preuss et al., 2011; Sisaphaithong et al.,2012 |
|  | *HvPHT1;2* | Roots, leaves | High | ↓AM/↑AM (weak) (depends on study) | Up-regulated by low Pi concentration. Sulphate, potassium or nitrogen deprivation reduce expression induced by low Pi. In transgenic rice *HvPHT1;2* promoter exhibits weak expression in leaves and strong in roots tissues (mainly in trichoblast cells and stele as well as in the sites of secondary roots emergence). *HvPHT1;2* promoter activity is reduced in older leaves. |  |
|  | *HvPHT1;3* | Roots | Low | AM-no influence | Up-regulated by low Pi, sulphate, potassium or nitrogen concentration as well as by high potassium concentration. AM and Pi influence tested using primers specific to both *HvPHT1;3* and *HvPHT1;4*. Higher expression of *HvPHT1;3* is associated with higher phosphate utilization efficiency (PUE). |  |
|  | *HvPHT1;4* | - | - | AM-no influence | Up-regulated by low Pi concentration. AM and Pi influence tested using primers specific to both *HvPHT1;3* and *HvPHT1;4*. |  |
|  | *HvPHT1;5* | No detectable | - | - | Not detected in roots, coleoptiles, young leaves, old leaves, leaf  sheaths, flag leaves, peduncles and ears. |  |
|  | *HvPHT1;6* | Root, leaf sheaths, old leaves, flag leaves, young leaves, peduncles, developing ears | Low (Km = 385± 61 *μ*M) | - | Very weak expression in roots, young leaves, peduncles  and developing ears. Strongly expressed in phloem cells of old leaves and flag leaves, so probably responsible for Pi remobilization. Expression in roots and shoots is up-regulated by low Pi concentration. Able to transport SO_4_^2-^ coupled to H^+^ as well as  NO_3_^-^ and Cl^-^. Higher expression of *HvPHT1;6* is associated with higher PUE. |  |
|  | *HvPHT1;7* | Not detected | - | - | Not detected in roots, coleoptiles, young leaves, old leaves, leaf  sheaths, flag leaves, peduncles and ears. |  |
|  | *HvPHT1;8* | Roots | - | ↑AM | Expression occurs only during AM symbiosis. |  |
|  | *HvPHT1;9* | Roots | - | - | Up-regulated by low Pi concentration. |  |
|  | *HvPHT1;10* | Roots | - | - | Up-regulated by low Pi concentration |  |
|  | *HvPHT1;11* | Roots | - | ↑AM | Not-detectable in non-mycorrhizal roots. |  |
| *Linum usitatissimum*/ flax | *LuPHT1;1* | Roots, stems, leaves | - | ↓AM | Expression independent on Pi concentration. | Walder et al., 2015 |
|  | *LuPHT1;2* | Roots, stems, leaves | - | ↓AM | Expression independent on Pi concentration. |  |
|  | *LuPHT1;3* | Roots, stems, leaves | - | ↓AM | Up-regulated by low Pi in non-mycorrhizal roots and down-regulated by low Pi in mycorrhizal roots. |  |
|  | *LuPHT1;4* | Roots, stems, leaves | - | ↓AM | Up-regulated by low Pi in non-mycorrhizal roots. |  |
|  | *LuPHT1;5* | Roots, leaves | - | ↑AM | Up-regulated by low Pi in mycorrhizal roots but not in non-mycorrhizal roots. |  |
|  | *LuPHT1;6* | No detectable | - | - | No detectable in any tested organs. |  |
|  | *LuPHT1;7* | Roots, stems, leaves | - | ↓AM | Up-regulated by low Pi in non-mycorrhizal roots and mycorrhizal roots. |  |
|  | *LuPHT1;8* | Roots, leaves | - | ↑AM | Expression independent on Pi concentration. |  |
|  | *LuPHT1;9* | - | - | - | No detectable in any tested organs. |  |
| *Lotus japonicus* | *LjPHT1;1* | Roots | - | ↓AM | - | Nakamori et al., 2002; Maeda et al., 2006; Volpe et al., 2016 |
|  | *LjPHT1;2* | Roots | - | ↓AM | - |  |
|  | *LjPHT1;3* | Roots | Low | ↑AM | Knockdown of *LjPHT1;3* causes reduction of AM colonization and Pi uptake during symbiosis as well as  necrotic root nodules. |  |
|  | *LjPHT1;4* | Roots | - | ↑AM | In non-mycorrhizal roots expressed in root tips and up-regulated by low Pi concentration. In mycorrhizal roots expressed in cortex cell containing arbuscules. Responsible for Pi sensing during lateral root development induced by low Pi supply. |  |
|  | *LjMPT* | - | - | - | Mitochondrial Pi transporter. |  |
| *Lupinus albus/*white lupin | *LaPHT1;1* | Roots, proteoid roots, stems, leaves, flowers | - | - | Induced by Pi deprivation. Weak expression in leaves and flowers. | Liu et al., 2001 |
|  | *LaPHT1;2* | Roots, proteoid roots, stems, leaves, flowers | - | - | Strongly expressed in normal and proteoid roots independently of Pi supply as well as under excess of Al and deficiencies of N, Mn, Fe. Really weak expression in stems, leaves and flowers. |  |
| *Lycopersion esculentum/*  tomato | *LePHT1;1* | Roots, hypocotyls, stems, cotyledons,  leaves, mature leaves, flowers, fruits (green and rape) | High (Km = 31μM) | ↓AM | Expressed in all root tissues (rhizodermis, trichoblasts, cortex, vascular cylinder, root cap). Expression up-regulated by sulphate deprivation. Low transcript level in cotyledons and hypocothyl. Up-regulated by Pi deficiency. Down-regulated in the mycorrhizal roots under low Pi but not high Pi supply conditions. | Daram et al., 1998; Liu C. et al., 1998; Rosewarne et al., 1999; Nagy et al., 2005; Poulsen et al., 2005; Xu et al., 2007; Chen et al., 2014 |
|  | *LePHT1;2* | Roots | - | ↓AM | Expression induced by Pi deprivation. In roots expressed in epidermis. No detectable in shoots organs. |  |
|  | *LePHT1;3* | Roots, stems, young leaves, flowers, fruits (green and rape) | High (proposed by Nagy et al., 2005) | ↑AM | Weak expression in flowers and fruits. Strongly up-regulated in mycorrhizal roots under low but not under high Pi supply. |  |
|  | *LePHT1;4* | Roots | Low (proposed by Nagy et al., 2005) | ↑AM | Strongly up-regulated in mycorrhizal roots during Pi-deficiency, but not under high Pi supply.  Mutant plants *lept4* exhibit Pi deficiency symptoms, reduced shoot biomass and altered roots morphology - shorter root system with very few laterals (14%  of the total number of WT laterals). |  |
|  | *LePHT1;5* | Roots, stems, young leaves, flowers, fruits (green and rape) | Low (proposed by Nagy et al., 2005) | ↑AM | The highest expression in rape fruits, in other organs low. Strongly up-regulated in mycorrhizal roots under low but not under high Pi supply. Does not express in roots under high Pi. |  |
|  | *LePHT1;6* | Roots, stems, young leaves | - | ↓AM | Weak expression in stems and leaves. |  |
|  | *LePHT1;7* | Roots, stems, young leaves, flowers, fruits (green and rape) | - | ↓AM | Weak expression in flowers and fruits, the highest expression in pi-starved roots. Does not express in roots and leaves under high Pi. |  |
|  | *LePHT1;8* | - | - | - | No detectable in all tested organs. |  |
|  | *LePHT2;1* | - | - | - | Putative chloroplast transporter, gene  extracted from GeneBank database. | Loth-Pereda et al., 2011 |
| *Medicago trunculata/* barrel medic | *MtPHT1;1* | Roots | Low (Km = 192 µM or  Km = 587 ± 60 µM - dependently on studies) | ↓AM | Expressed in epidermal and cortex cells. Up-regulated during Pi-starvation. | Liu H. et al., 1998;  Chiou et al., 2001; Harrison et al., 2002; Karandashov et al., 2004; Xiao et al., 2006; Javot et al., 2007a; Liu et al., 2008; Grunwald et al., 2009; Balzergue et al., 2013;Volpe et al., 2016; |
|  | *MtPHT1;2* | Roots | Low (Km = 641 ± 64 µM) | ↓AM | Strongly expressed in central cylinder, but also in cortex and epidermal cells. Up-regulated during Pi-starvation. |  |
|  | *MtPHT1;3* | Roots | Low (Km = 858 ± 91 µM) | ↓AM (with exceptions) | Expressed exclusively in vascular cylinder. Up-regulated during Pi-starvation. |  |
|  | *MtPHT1;4* | Roots | Low  (Km = 493 or Km= 668 µM - measured in two different yeast mutants) | ↑AM | In non-mycorrhizal roots expressed in root tips and up-regulated by low Pi concentration. In mycorrhizal roots expressed in cortex cell containing arbuscules. It is responsible for Pi sensing during lateral root development induced by low Pi supply and it is required for symbiosis formation. |  |
|  | *MtPHT1;5* | - | High (Km = 13 ± 2 µM) | ↓AM (with exceptions) | Up-regulated during Pi-starvation. |  |
|  | *MtPHT1;6* | - | - | ↓AM | - |  |
|  | *MtPHT2;1* | - | - | - | Putative chloroplast PHT2 gene. Sequence available in GeneBank database. | Loth-Pereda et al., 2011 |
| *Nicotiana tabacum/* tabacco | *NtPHT1;1* | Roots, stems, immature leaves,  mature leaves, old leaves. | - | ↓AM (leaves), AM-no influence (roots) | Expression in plant organs analysed by northern blotting with probe specific for both NtPHT1;1 and NtPHT1;2. Up-regulated by Pi deficiency in roots and leaves. | Kai et al., 2002; Chen et al., 2007 |
|  | *NtPHT1;2* | Roots (stems, immature leaves,  mature leaves, old leaves) | - | ↓AM | In roots induced by Pi-depletion. Expression in plant organs analysed by northern blotting with probe specific for both NtPHT1;1 and NtPHT1;2 showed expression in above-ground organs, but RT-PCR reaction with specific primers did not detected the gene expression in the leaves. |  |
|  | *NtPHT1;3* | Roots, leaves | - | ↑AM | In roots expression enhanced by AM formation. In leaves independent on Pi concentration and AM formation. |  |
|  | *NtPHT1;4* | Roots, leaves | - | ↑AM | In roots induced by AM formation. Weak expression in the leaves during AM symbiosis, independently on Pi supply. |  |
|  | *NtPHT1;5* | Roots | - | ↑AM | Induced by AM formation. |  |
|  | *NtPHT2;1* | Leaves | - | ↓AM | - |  |
| *Petunia* x *hybrida/*petunia | *PhPHT1;1* | Roots | - | AM-non influence | Constitutively expressed. | Wegmüller et al., 2008; Breuillin et al., 2010 |
|  | *PhPHT1;2* | Roots | - | AM-no influence/↓AM (weak) | Strongly repressed by high Pi level. |  |
|  | *PhPHT1;3* | Roots | - | ↑AM | Expression in symbiotic roots inhibited during high Pi treatment. |  |
|  | *PhPHT1;4* | Roots | - | ↑AM | Specifically induced by AM fungi but inhibited in symbiotic roots during high Pi treatment. |  |
|  | *PhPHT1;5* | Roots | - | ↑AM | Strongly repressed by high Pi, also in symbiotic roots. |  |
|  | *PhPHT1;7* | Roots | - | - | Strongly repressed by high Pi. |  |
| *Phaseolus vulgaris*/common bean | *PvPHT1;1* | Roots, shoots | - | - | Specifically induced under Pi deficiency | Tian et al., 2007 |
| *Physcomitrella*  *Patens*/ spreading earthmoss (moss) | *PpPHT2;1* | - | - | - | Putative chloroplast PHT2 gene. Sequence available in GeneBank database. | Loth-Pereda et al., 2011 |
| *Platanus acerifolia/* London planetree | *PaPHT1* | - | - | - | Putative gene extracted from the NCBI GeneBank database. | Loth-Pereda et al., 2011 |
| *Poncirus trifoliata/*hardy orange | *PtaPHT1;1* | Roots | - | ↓AM |  | Shu et al., 2012 |
|  | *PtaPHT1;2* | Roots | - | ↓AM |  |  |
|  | *PtaPHT1;3* | Roots | - | ↓AM |  |  |
|  | *PtaPHT1;4* | Roots | - | ↑AM |  |  |
|  | *PtaPHT1;5* | Roots | - | ↑AM |  |  |
|  | *PtaPHT1;6* | Roots | - | ↓AM | Expressed independently of Pi concentration |  |
|  | *PtaPHT1;7* | Roots | - | ↓AM |  |  |
| *Populus deltoides/* eastern cottonwood | *PdPHT1;1-PdPHT1;12* | Roots | - | - | - | Loth-Pereda et al., 2011 |
| *Populus trichocarpa/* black cottonwood | *PtPHT1;1* | Adventitious  roots, central cylinder, petioles, mature leaves, senescing leaves,  female inflorescences | Low (based on lack of yeast mutant complementation) | ↓AM, ↓ECM | Up-regulated in senescing leaves. Expression after fungi colonization was analysed on *Populus tremula* x *Populus alba* roots. | Loth-Pereda et al., 2011 |
|  | *PtPHT1;2* | Adventitious  roots, central cylinder, petioles, mature leaves, senescing leaves, male and female inflorescences | Low (based on lack of yeast mutant complementation). | AM and ECM-no influence | Weak expression in female inflorescences. Up-regulated in senescing leaves. Expression after fungi colonization was analysed on *Populus tremula* x *Populus alba* roots. |  |
|  | *PtPHT1;3* | Adventitious  roots, central cylinder, petioles, mature leaves, senescing leaves | - | ↑AM, ECM-no influence | Expression after fungi colonization was analysed on *Populus tremula* x *Populus alba* roots. |  |
|  | *PtPHT1;4* | Adventitious  roots, central cylinder, petioles, mature leaves, senescing leaves, male and female inflorescences | Low (based on lack of yeast mutant complementation). | ↑AM, ECM-no influence | Expression analysed together with *PtPHT1;7.* Expression after fungi colonization was analysed on *Populus tremula* x *Populus alba* roots. |  |
|  | *PtPHT1;5* | Adventitious  roots, central cylinder, petioles, mature leaves, senescing leaves, male and female inflorescences | - | ↑AM, ECM-no influence | In roots down-regulated at low-Pi conditions. Up-regulated in senescing leaves. Expression after fungi colonization was analysed on *Populus tremula* x *Populus alba* roots. |  |
|  | *PtPHT1;6* | Adventitious  roots, central cylinder, petioles, mature leaves, senescing leaves, male inflorescences | High (based on yeast mutant complementation) | AM and ECM-no influence | In roots down-regulated at low-Pi conditions. Up-regulated in senescing leaves. Expression after fungi colonization was analysed on *Populus tremula* x *Populus alba* roots. |  |
|  | *PtPHT1;7* | Adventitious  roots, central cylinder, petiosle, mature leaves, senescing leaves, male and female inflorescences | - | ↑AM, ECM-no influence | Expression analysed together with *PtPHT1;4*. Expression after fungi colonization was analysed on *Populus tremula* x *Populus alba* roots. |  |
|  | *PtPHT1;8* | - | - | - | No-detectable in any tested organs. |  |
|  | *PtPHT1;9* | Adventitious  roots, central cylinder, petioles, mature leaves, senescing leaves | Low (based on lack of yeast mutant complementation) | ↑AM ↑ECM | Up-regulated in senescing leaves. Expression after fungi colonization was analysed on *Populus tremula* x *Populus alba* roots. |  |
|  | *PtPHT1;10* | Roots | Low (based on lack of yeast mutant complementation) | ↑AM | Specifically induced by AM formation. |  |
|  | *PtPHT1;11* | Adventitious  roots, central cylinder, petioles, mature leaves, senescing leaves | - | ↓AM ↓ECM | Down-regulated in senescing leaves. Expression after fungi colonization was analysed on *Populus tremula* x *Populus alba* roots. |  |
|  | *PtPHT1;12* | Adventitious  roots, central cylinder, petioles, mature leaves, senescing leaves | - | ↑AM ↑ECM | Up-regulated in senescing leaves. Expression after fungi colonization was analysed on *Populus tremula* x *Populus alba* roots. |  |
|  | *PtPHT2;1* | - | - | - | Putative chloroplast *PHT2* gene. |  |
|  | *PtPHT2;2* | - | - | - | Putative chloroplast *PHT2* gene. |  |
| *Pteris vitatta/*Chinese ladder brake (fern) | *PvPHT1;1* | - | Moderate complementation of yeast mutant | - | RNA isolated from liquid-culture-grown gametophytes. | DiTusa et al., 2016 |
|  | *PvPHT1;2* | - | Moderate complementation of yeast mutant | - | RNA isolated from liquid-culture-grown gametophytes. |  |
|  | *PvPHT1;3* | - | Able to complement yeast mutant. | - | High affinity arsenate transporter. Expression induced by arsenate and low Pi level.  RNA isolated from liquid-culture-grown gametophytes. |  |
| *Ricinus communis/* castorbean | *RcPHT1;1 – RcPHT1;6* | - | - | - | Putative *PHT1* genes extracted from the NCBI GeneBank database. | Loth-Pereda et al., 2011 |
| *Sesbania rostrata* | *SrPHT1* | - | - | - | Putative *PHT1* extracted from the NCBI GeneBank database. | Davies et al., 2002;  Loth-Pereda et al., 2011 |
|  | *SrPHT1;2* | - | - | - |  |  |
| *Setaria italica*/foxtail millet | *SiPHT1;1* | Shoots | - | - | - | Ceasar et al., 2014 |
|  | *SiPHT1;2* | Roots, shoots, old leaves | - | ↑AM/↓AM (dependently on plant organ) | Expressed in all growth conditions. In roots weakly down-regulated, in leaves up-regulated around 2 times during AM symbiosis. In leaves expression is around 7 times higher under low Pi conditions compared to high Pi. |  |
|  | *SiPHT1;3* | Roots, shoots, old leaves | - | ↓AM (weakly) | Strongly expressed in leaves under high and low Pi but weakly down-regulated by AM symbiosis. Weak expression in roots. |  |
|  | *SiPHT1;4* | Roots, shoots, old leaves | - | AM-independent expression in the roots | Highly expressed in roots, especially under low Pi concentration. |  |
|  | *SiPHT1;5* | Not detected | - | - | - |  |
|  | *SiPHT1;6* | Shoot, old leaves | - | - | Highly expressed in old leaves during Pi starvation but not under high Pi growth conditions. |  |
|  | *SiPHT1;7* | Not detected | - | - | - |  |
|  | *SiPHT1;8* | Roots, shoot | - | ↑AM | Specifically induced by AM in roots, in shoot expressed during Pi starvation. |  |
|  | *SiPHT1;9* | Roots, shoot | - | ↑AM | Specifically induced by AM in roots, in shoot expressed during Pi starvation. |  |
|  | *SiPHT1;10* | Shoots | - | - | Expressed during Pi starvation, no detectable under high Pi growth conditions. |  |
|  | *SiPHT1;11* | Shoots, leaves | - | ↓AM (weakly in the leaves) | Highly expressed in old leaves during Pi starvation but not under high Pi growth conditions. |  |
|  | *SiPHT1;12* | Roots, shoots, leaves | - | ↑AM | Highly expressed in old leaves during Pi starvation but not under high Pi growth conditions. Weak expression in the roots specifically induced by AM symbiosis. |  |
| *Solanum melongena/*eggplant | *SmPHT1;1* | Roots, leaves | - | - | Up-regulated by Pi deficiency in roots and leaves. | Chen et al., 2007 |
|  | *SmPHT1;2* | Roots | - | ↑AM | Strongly up-regulated by Pi-depletion in non-mycorrhizal roots. During symbiosis expressed also under high Pi condition. |  |
|  | *SmPHT1;3* | Roots, leaves | - | ↑AM | In Pi-deprived roots expression strongly enhanced by AM formation. In leaves weak expression independent on Pi supply and AM. |  |
|  | *SmPHT1;4* | Roots | - | ↑AM | In non-AM roots expressed weakly under Pi-starvation. |  |
|  | *SmPHT1;5* | Roots | - | ↑AM | In non-AM roots expressed weakly under Pi-starvation. |  |
|  | *SmPHT2;1* | Leaves | - | AM-no influence | Not expressed in roots. In leaves expression independent on Pi supply and AM formation. |  |
| *Solanum tuberosum/*potato | *StPHT1;1* | Roots, growing tubers,  sprouting tubers, stolons, stems, petioles, young leaves, old leaves, floral buds, open flower, sepals, petals, stamens, carpels | Low (Km = 280 µM) | ↓AM | Constitutively expressed in roots, independently on Pi supply. Under optimal nutrition conditions expressed at a lower level in growing tubers, sprouting tubers, and flowers and not expressed in stem or young leaves. Under Pi deprivation expressed in all tested organs (but weakly in the stem and young leaves). In roots expressed also under sulphur, nitrogen and potassium deprivation conditions. | Leggewie et al., 1997; Raush et al., 2001; Gordon-Weeks et al., 2003; Karandashov et al., 2004; Raush et al., 2004; Nagy et al., 2005; |
|  | *StPHT1;2* | Roots, stolons developing tubers | Low (Km = 130 µM) | ↓AM | Strongly expressed in roots. Expression inducible by Pi deprivation and weakly by sulphate deprivation, down-regulated by high Pi.  Exhibit polar localization- only in apical surface of plasma membrane of epidermal cells. No- detectable in shoots organs. |  |
|  | *StPHT1;3* | Roots, leaves | Low (Km = 64 ± 3 µM) | ↑AM | Described as mycorrhizal specific transporter, but basal expression was also detected in non-mycorrhizal roots. Weakly expressed in leaves of plants infected by AM fungi. |  |
|  | *StPHT1;4* | Roots | Low | ↑AM | Expressed only in mycorrhizal roots.  No-detectable in shoots organs. |  |
|  | *StPHT1;5* | Roots | Low | ↑AM | Expressed only in mycorrhizal roots. No-detectable in shoots organs. |  |
|  | *StPHT2;1* | Stems, young leaves, mature leaves, petioles, floral buds, open flowers, sepals, petals, stamens, carpels | - | - | Protein localized in chloroplast. The stronger gene expression occurs in young leaves and flowers. In leaves is clearly down-regulated during senescence. Expression is induced by light. |  |
| *Sorghum bicolor/*sorghum | *SbPHT1;1* | Roots | - | ↓AM | Up-regulated by low Pi concentration in non-mycorrhizal roots. | Sisaphaithong et al. 2012; Walder et al., 2015 |
|  | *SbPHT1;2* | Roots, stems, leaves, stamens, pistils | - | ↓AM | Expression independent on Pi concentration. |  |
|  | *SbPHT1;3* | - | - | - | Not detectable in any tested organs. |  |
|  | *SbPHT1;4* | Roots, stems, leaves | - | AM-no influence | Expression independent on Pi concentration. |  |
|  | *SbPHT1;5* | Roots, stamens | - | AM-no influence | Expression independent on Pi concentration. |  |
|  | *SbPHT1;6* | Roots, stems, leaves, stamens, pistils | - | ↓AM | Down-regulated by low Pi concentration. |  |
|  | *SbPHT1;7* | Roots, stems, leaves, stamens, pistils | - | AM-no influence | Expression independent on Pi concentration. |  |
|  | *SbPHT1;8* | Roots, stamens | - | ↑AM | Up-regulated by low Pi concentration in non-mycorrhizal roots. |  |
|  | *SbPHT1;9* | Roots, leaves | - | ↑AM | Ortholog of rice *OsPHT1;13.* Name based on: Walder et al., 2015. In Sisaphaithong et al. 2012 named SbPT1. |  |
|  | *SbPHT1;10* | Roots | - | ↑AM | Ortholog of rice *OsPHT1;13.* Name based on: Walder et al., 2015. In Sisaphaithong et al. 2012 named SbPT2. |  |
|  | *SbPHT1;11* | Roots, stamens, pistils | - | ↑AM | Strongly up-regulated in mycorrhizal roots. Ortholog of rice *OsPHT1;11.* Name based on: Walder et al., 2015. In Sisaphaithong et al. 2012 named SbPT1. |  |
| *Thinopyrum*  *elongatum* (*Agropyron elongatum*)*/* tall wheatgrass | *TePHT1;2* | Roots, leaves | - | - | Expressed under high Pi supply, undetectable under low Pi. | Davies et al., 2002 |
|  | *TePHT1;3* | Roots, leaves | - | - | Constitutively expressed, stronger in roots and weaker in leaves. |  |
|  | *TePHT1;6* | - | - | - | No-detectable |  |
| *Thinopyrum intermediu/*intermediate wheatgrass | *TiPHT1;2* | Roots, leaves | - | - | Strongly expressed under high Pi supply, in leaves undetectable under low Pi. | Davies et al., 2002 |
|  | *TiPHT1;3* | Roots, leaves | - | - | Constitutively expressed, stronger in roots and weaker in leaves. |  |
|  | *TiPHT1;6* | - | - | - | No-detectable |  |
| *Triticum aestivum/*common wheat | *TaPHT1;1* | - | - | - | - | Davies et al., 2002; Glassop et al., 2005; Tittarelli et al., 2007; Miao et al., 2009; Sisaphaithong et al., 2012; Guo et al., 2013; Liu et al., 2013; Aziz et al., 2014; Guo et al., 2014; Duan et al., 2015 |
|  | *TaPHT1;2* | Roots, stems, young leaves, mature leaves | High (Km = 23,6 µM) | ↓AM | More abundant in P-efficient genotypes than in P-inefficient genotypes.  In some wheat variety expression in roots independent on P-supply, in other induced by P-deprivation (sugars enhance, cytokinins and N-deficiency inhibit gene induction by P-starvation). In leaves undetectable under high Pi conditions. During Pi deprivation, overexpression increased plant dry mass and photosynthetic  efficiencies. |  |
|  | *TaPHT1;3* | Roots, leaves | - | - | In roots expression independent on Pi supply, in leaves up-regulated by low Pi concentration. |  |
|  | *TaPHT1.4* | Roots | High (Km= 35,3 μM) | ↓AM or without influence | Expressed following a diurnal pattern. Overexpression significantly improved growth traits under low-Pi conditions |  |
|  | *TaPHT1;5* | Roots, stems, young leaves, mature leaves | - | - | In experimental wheat line 81(85)-5-3-3-3 were detected two isoforms of *TaPHT1;5*:  *TaPT5;1* and *TaPT5;2*. *TaPT5;1* is constitutively expressed in roots and leaves, *TaPT5;2* only in Pi starved roots. |  |
|  | *TaPHT1;6* | Roots, leaves |  | - | Up-regulated by Pi deficiency. |  |
|  | *TaPHT1;7* | Roots | - | - | - |  |
|  | *TaPHT1;8* | Roots, stems, young leaves, mature leaves | - | ↑AM | Name also *TaPHT1;myc*. Not expressed in non-mycorrhizal roots |  |
|  | *TaPHT1;9* | - | - | - | - |  |
|  | *TaPHT1;10* | Roots | - | ↑AM | Strongly expressed in mycorrhizal roots. |  |
|  | *TaPHT1;11* | Roots | - | ↑AM | Strongly expressed in mycorrhizal roots. |  |
|  | *TaPHT1;12* | Roots | - | ↑AM | Strongly expressed in mycorrhizal roots. |  |
|  | *TaPHT2;1* | Roots, stems, young leaves, mature leaves | Low (Km = 225 µM) | - | Located in chloroplasts envelope, transfers Pi from cytosole to chloroplast. Exhibits a  circadian rhythmic expression pattern. Weak expression in root tissues. Regulates photosynthetic capacities, Pi homeostasis and wheat  growth under various Pi supply conditions. |  |
|  | *TaPHT3;1* | Roots, stems, young leaves, mature leaves | - | - | - |  |
| *Triticum monococcum/* einkorn wheat | *TmPHT1* | - | - | - | Putative *PHT1* extracted from the NCBI GeneBank database. | Loth-Pereda et al., 2011 |
| *Vitis vinifera/*common grape vine | *VvPHT1* | - | - | - | Putative *PHT1* extracted from the NCBI GeneBank database. | Loth-Pereda et al., 2011 |
| *Zea mays/*corn | *ZmPHT1;1* | Roots, stems, young leaves, old leaves, anthers, silk, young seeds, germinating seeds cob | - | ↓AM | Pi deficiency has no influence on *ZmPHT1;1* expression in roots. | Wright et al., 2005; Glassop et al., 2005; Nagy et al., 2006; Tian et al., 2013; Willmann et al., 2013; Liu F. et al., 2016 |
|  | *ZmPHT1;2* | Roots, stems, young leaves, old leaves, anthers, silk, young seeds, cob | - | ↑AM or no influence (dependently on studies) | Expression in roots up-regulated by Pi deficiency. |  |
|  | *ZmPHT1;3* | Roots, stems, young leaves, old leaves, anthers, silk, young seeds, cob | - | ↓AM or no-influence (dependently on studies) | Expression in roots up-regulated by Pi deficiency. Highly expressed in pollen. |  |
|  | *ZmPHT1;4* | Roots, stems, young leaves, old leaves, anthers, silk, young seeds, cob | - | ↑AM | Weak expression in cob. |  |
|  | *ZmPHT1;5* | Roots | - | ↓AM | Expression up-regulated by Pi deficiency. |  |
|  | *ZmPHT1;6* | Roots, leaves |  | ↑AM | In roots and leaves expression strongly up-regulated by Pi deficiency. Expression in old leaves higher than in young ones. Ortholog of rice *OsPHT1;11.* AM symbiosis upregulate gene expression around 100 times |  |
|  | *ZmPHT1;7* | Roots | - | ↑AM | Expression up-regulated by Pi deficiency. |  |
|  | *ZmPHT1;8* | Roots, leaves | - | ↓AM | Expression in roots up-regulated by Pi deficiency |  |
|  | *ZmPHT1;9* | Roots, stems, leaves, embryo, germinating seeds | - | ↑AM | Expression in roots up-regulated by Pi deficiency |  |
|  | *ZmPHT1;10* | Roots | - | AM - no influence | Expression up-regulated by Pi deficiency. |  |
|  | *ZmPHT1;11* | Roots | - | ↑AM | AM symbiosis up-regulate gene expression around 160 times |  |
|  | *ZmPHT1;12* | Roots | - | AM - no influence | Expression up-regulated by Pi deficiency. |  |
|  | *ZmPHT1;13* | Roots, stems, germinating seeds | - | ↓AM | Expression in roots up-regulated by Pi deficiency |  |

^a^ Description of rice and *Arabidopsis* proteins in main body of the publication.

^b^ Pi transporters and their genes are named differently according to publications. For example *Medicago truncatula* gene *MtPHT1;2* may be also named *MtPT2* or *MEDtr;Pht1;2* when *Hordeum vulgare* gene *HvPHT1;1* may be called *HORvu;Pht1;1* or *HvPT1* etc.

^c^ high affinity – up to 50 μM; low affinity – above 50 μM

^d^ ↓AM- down-regulated by arbuscular mycorrhizal symbioses, ↑AM- up-regulated by arbuscular mycorrhizal symbioses, ↓ECM- down-regulated by aectomycorrhizal symbioses, ↑ECM- up-regulated by ectomycorrhizal symbioses

^e^ List of references in main body of the publication.
